# The role of psychosocial well-being and emotion-driven impulsiveness in food choices of European adolescents

**DOI:** 10.1186/s12966-023-01551-w

**Published:** 2024-01-02

**Authors:** Stefanie Do, Vanessa Didelez, Claudia Börnhorst, Juul M.J. Coumans, Lucia A. Reisch, Unna N. Danner, Paola Russo, Toomas Veidebaum, Michael Tornaritis, Dénes Molnár, Monica Hunsberger, Stefaan De Henauw, Luis A. Moreno, Wolfgang Ahrens, Antje Hebestreit, on behalf of the IDEFICS/I.Family consortia

**Affiliations:** 1 Department of Epidemiological Methods and Etiological Research, Leibniz Institute for Prevention Research and Epidemiology - BIPS, Achterstrasse 30, 28359 Bremen, Germany; 2 Department of Biometry and Data Management, Leibniz Institute for Prevention Research and Epidemiology - BIPS, Bremen, Germany; 3 Faculty of Mathematics and Computer Science, University of Bremen, Bremen, Germany; 4 Teaching and Learning Centre, Open University of the Netherlands, Heerlen, The Netherlands; 5 Cambridge Judge Business School, University of Cambridge, Cambridge, UK; 6 Department of Clinical Psychology, Altrecht Eating Disorders Rintveld, Utrecht University, Zeist, The Netherlands; 7 Institute of Food Sciences, National Research Council, Avellino, Italy; 8 Department of Chronic Diseases, National Institute for Health Development, Tallinn, Estonia; 9 Research and Education Institute of Child health, REF, Strovolos, Cyprus; 10 Department of Pediatrics, Medical School, University of Pécs, Pécs, Hungary; 11 School of Public Health and Community Medicine, Institute of Medicine, University of Gothenburg, Gothenburg, Sweden; 12 Department of Public Health, Faculty of Medicine and Health Sciences, Ghent University, 9000 Ghent, Belgium; 13 GENUD (Growth, Exercise, Nutrition and Development) Research Group, Instituto Agroalimentario de Aragón (IA2), Centro de Investigación Biomédica en Red Fisiopatología de la Obesidad y Nutrición (CIBERObn), Instituto de Investigación Sanitaria Aragón (IIS Aragón), University of Zaragoza, 50009 Zaragoza, Spain

**Keywords:** Causal mediation, Food preferences, I.Family study, Impulsivity, Mental health, Causal inference, Adolescents

## Abstract

**Background:**

It is unclear whether a hypothetical intervention targeting either psychosocial well-being or emotion-driven impulsiveness is more effective in reducing unhealthy food choices. Therefore, we aimed to compare the (separate) causal effects of psychosocial well-being and emotion-driven impulsiveness on European adolescents’ sweet and fat propensity.

**Methods:**

We included 2,065 participants of the IDEFICS/I.Family cohort (mean age: 13.4) providing self-reported data on sweet propensity (score range: 0 to 68.4), fat propensity (range: 0 to 72.6), emotion-driven impulsiveness using the UPPS-P negative urgency subscale, and psychosocial well-being using the KINDL^R^ Questionnaire. We estimated, separately, the average causal effects of psychosocial well-being and emotion-driven impulsiveness on sweet and fat propensity applying a semi-parametric doubly robust method (targeted maximum likelihood estimation). Further, we investigated a potential indirect effect of psychosocial well-being on sweet and fat propensity mediated via emotion-driven impulsiveness using a causal mediation analysis.

**Results:**

If all adolescents, hypothetically, had high levels of psychosocial well-being, compared to low levels, we estimated a decrease in average sweet propensity by 1.43 [95%-confidence interval: 0.25 to 2.61]. A smaller effect was estimated for fat propensity. Similarly, if all adolescents had high levels of emotion-driven impulsiveness, compared to low levels, average sweet propensity would be decreased by 2.07 [0.87 to 3.26] and average fat propensity by 1.85 [0.81 to 2.88]. The indirect effect of psychosocial well-being via emotion-driven impulsiveness was 0.61 [0.24 to 1.09] for average sweet propensity and 0.55 [0.13 to 0.86] for average fat propensity.

**Conclusions:**

An intervention targeting emotion-driven impulsiveness, compared to psychosocial well-being, would be marginally more effective in reducing sweet and fat propensity in adolescents.

**Supplementary Information:**

The online version contains supplementary material available at 10.1186/s12966-023-01551-w.

## Introduction

Food choices and eating behaviors are shaped by environmental factors during childhood and adolescence [1, 2]. Eating unhealthy foods, such as sweet and/or fatty foods, in response to negative emotions is a maladaptive emotion regulation strategy [3, 4]. Recent research suggests that sweet and fat taste preference is the direct effect of high-sugar and high-fat foods on neurobehavioral adaptations independent of obesity or metabolic parameters [5]. The consumption of these foods lead to greater activation of the brain reward system stimulating hedonic, reinforcing, and motivational processes [3].

Psychological factors like emotional well-being and psychological traits may play an important role in food choices. Emotional well-being presents a multidimensional composite that encompasses how positive an individual feels generally and about life overall [6] and is associated with different health behaviors [3, 7]. While an increasing amount of studies suggests a positive association between emotional well-being and healthy food choices [8]; only one study in adults reported that higher levels of emotional well-being were associated with fewer unhealthy food choices [9]. Research on emotional well-being in youth has been scarce and focused particularly on the dimension of psychosocial well-being which emphasizes intra- and interpersonal levels of positive functioning in youth [10]. Personality traits linked to impulsive behaviors may act as potential mediators between psychosocial well-being and unhealthy food choices. Lower psychosocial well-being is associated with higher levels of negative emotions [11], and may hence increase the risk of acting impulsively, which, in turn may be a pathway to unhealthy food choices. For instance, emotion-driven impulsiveness, which is the tendency to act impulsively in response to negative emotions [12], was previously reported to be associated with lower levels of psychosocial well-being [9, 13] as well as with consuming more energy-dense snacks in adolescents [14].

However, the precise roles of psychosocial well-being and emotion-driven impulsiveness in shaping food choices in adolescents are not clear: Most of the studies were mainly conducted in adults and investigated the associations of either psychosocial well-being or emotion-driven impulsiveness on food choices [9, 14, 15]; while only one study applied a causal mediation analysis [9]. Recent methodological advances of potential outcomes approaches enable estimating causal effects that may provide practically relevant information on interventions needed to reduce unhealthy food choices. These methods provide further benefits by minimizing the assumptions such as linearity on the distribution of the data [16] or allowing interactions between the exposure and mediator [17]. For informing the development of effective interventions, we aimed to answer the following key question: (Q1) Is increasing psychosocial well-being or decreasing emotion-driven impulsiveness more promising to improve food choices among European adolescents? Formally, (Q1) is asking for the separate causal effects of psychosocial well-being and emotion-driven impulsiveness on sweet and fat propensity.

With regards to a potential mediating role of emotion-driven impulsiveness in the relationship between adolescents’ psychosocial well-being and food choices, we considered the following additional question: (Q2) How much, if at all, is the effect of psychosocial well-being on adolescents’ food choices mediated by emotion-driven impulsiveness? Formally, (Q2) is asking for the direct and indirect effects of psychosocial well-being on sweet and fat propensity mediated by emotion-driven impulsiveness. The causal nature of questions (Q1) and (Q2) means that we aim to assess how sweet and fat propensity would change under hypothetical interventions on psychosocial well-being and emotion-driven impulsiveness. While (Q2) may not actually have any additional practical implications over and above (Q1), it may provide a deeper understanding of psychological mechanisms and help (re)interpret previous study findings.

## Methods

### Data source

This study is based on the IDEFICS/I.Family cohort, a prospective multi-center study that investigated the role of health-related behaviors and their determinants in the development of obesity and metabolic disorders in youth in eight European countries: Belgium, Cyprus, Estonia, Germany, Hungary, Italy, Spain, and Sweden [18]. At total of 16,230 children aged 2 to 9 years participated at baseline (W1: 2007–2008). Of those, 13,587 children participated in the second wave (W2: 2009–2010). The third wave (W3: 2013–2014) re-examined 7,123 index children aged 7 to 17 years [18]. Index children were those already participating in W1 and/or W2. The fourth and most recent wave (W4) was conducted in 2020–2021 as an online survey. Even though the survey was conducted during the Covid-19 pandemic, participants were asked to report behavior from the time before the pandemic; by the time of this publication 5,073 participants were enrolled. At all study waves, informed consent was either obtained from adolescents (≥ 12 years) or from parents who gave their consent for themselves and their younger children (< 12 years). The cohort study was performed according to the standards of the Declaration of Helsinki. Ethical approval was obtained from the respective ethics committees by all eight study centers according to local standards.

### Sweet and fat propensity

The score for sweet propensity was calculated as the sum of the weekly frequency of intake of food and drink items with high sugar content divided by the total frequency of all food and drink items included in the Food Frequency Questionnaire (FFQ), and multiplied by 100. The FFQ has been previously validated and tested for reproducibility [19–21]. Likewise, the score for fat propensity was calculated as the proportion of the consumption of high-fat foods. The observed scores ranged from 0 to 68.4 for sweet propensity and 0 to 72.6 for fat propensity. A value of 50 for the sweet or fat propensity indicates that half of the reported food consumption frequencies included foods rich in sugar or high in fatty content, respectively. A complete list of the food and drink items included in the sweet and fat propensity is available in the supplementary materials (Additional file 1).

#### Emotion-driven impulsiveness

The negative urgency subscale from the Urgency, Premeditation, Perseverance, Sensation seeking, and Positive urgency (UPPS-P) questionnaire [22] was used to operationalize emotion-driven impulsiveness. The negative urgency subscale included twelve items on a four-point Likert scale ranging from ‘1’ (strongly agree) to ‘4’ (strongly disagree). All items of the original scale, except for one item, were inversely coded to allow all items to run in the same direction. The scale score was then calculated as the sum of item responses, with higher scores indicating more impulsive tendencies when experiencing negative emotions (range: 0 to 48). The validity and reliability of the UPPS-P have been shown elsewhere [23]. The internal consistency in this study was good with a Cronbach’s alpha of 0.85. To ease the interpretation, we categorized the negative urgency score into tertiles: The ‘low’ exposure category ranges from 12 to 20 points (N = 709), the ‘moderate’ exposure category from 21 to 28 points (N = 741), and the ‘high’ exposure category from 29 to 48 points (N = 615).

#### Psychosocial well-being

Study participants completed 16 items of the four subscales of the KINDL^R^ Questionnaire that was specifically developed to measure Health-Related Quality of Life in children and adolescents [24]. The four subscales, emotional health, self-esteem, family life, and relations to friends have four items each. Survey centers were asked to use the translated English or local language version as the original version was developed in German. The 16 items were scored on a five-point Likert scale ranging from ‘0’ (Never) to ‘4’ (All the time). Since response categories differed between W1/W2 and W3/W4, we deviated from the original scoring and assigned three points to ‘Often/All the time’ (W1/W2) and ‘Often’ and ‘All the time’ (W3/W4), respectively. Six items of the original scale were inversely coded, allowing the items to run in the same direction. All items were summed by subscale to create a total sum score, with higher values indicating higher psychosocial well-being (range: 0 to 48). The validity and reliability of the KINDL^R^ have been shown elsewhere [25]. The set of items in this study showed a Cronbach’s alpha of 0.46, indicating moderate internal consistency. We divided the well-being score into tertiles with varying group size: The ‘low’ exposure category ranges from 9 to 35 points (N = 594), the ‘moderate’ exposure category from 36 to 40 points (N = 733), and the ‘high’ exposure category from 41 to 48 points (N = 738).

### Covariates

We selected suitable covariates to adjust for confounding in the different causal analyses (i.e. for each exposure-outcome pair and for the causal mediation analysis). This was guided by directed acyclic graphs (DAGs) constructed according to subject-matter expertise and previous empirical research, where we employed a framework for dietary behavior to identify relevant variables [26] (Additional file 2). Based on this framework we included sex (0 = male / 1 = female) and age (in years). Countries of recruitment were grouped as Central Europe (Belgium, Germany, Hungary), Northern Europe (Estonia, Sweden), and Southern Europe (Italy, Spain). The highest educational level of the parents was determined according to the International Standard Classification of Education (ISCED) and categorized as follows: 0 to 2 = low, 3 to 5 = moderate, 6 to 8 = high [27]; the ‘low’ category was then combined with the ‘moderate’ category due to the low prevalence of low education. The BMI z-score, calculated from measured height and weight, was derived according to the extended International Obesity Task Force criteria [28] and children were classified as thin/normal weight versus overweight/obesity. Self-reported physical activity was assessed as sports club membership (0 = no / 1 = yes), which was shown to be a useful proxy in previous studies [29]. A proxy variable for sleep quality was assessed via the self-reported sleep characteristics bedtime routine, trouble getting up in the morning, and difficulties falling asleep. These sleep characteristics were added up and ranged from ‘0’ to ‘2’ after combining categories 2 and 3 due to low frequencies in this study; individuals scoring 3 were considered as having poor sleep quality. Media use was measured as exposure to audiovisual media (PC and TV) in hours per week. Additionally, we adjusted for the previous well-being score as well as the previous scores for sweet and fat propensity.

### Analysis group

All relevant variables are available in all waves of the IDEFICS/I.Family cohort with the exception of emotion-driven impulsiveness that was only measured in W3 and W4. The present study used variables measured at W2, W3, and W4 with sweet and fat propensity as the dependent variable and the remaining variables as the independent variables. However, time intervals between W3 and W4 were up to 7 years. So, we conducted our main analyses allowing for contemporaneous effects at W3, i.e. assuming each questionnaire to reflect exposure during the preceding period. In one of our sensitivity analyses we allowed for time-delayed effects with the exposure variable measured at W3 and the mediator and outcome variables at W4. Hence, the present study uses mainly data from W3 and excluded adolescents from Cyprus, whose data for W4 were unavailable at the time of analysis (N = 498). The analysis group included adolescents aged ≥ 10 years providing complete information on the main variables in W3 (or W4 in our sensitivity analyses): (i) psychosocial well-being, (ii) emotion-driven impulsiveness (when at least eight items from the UPPS-P were completed), (iii) sweet propensity, and (iv) fat propensity. Furthermore, we excluded adolescents diagnosed with mental disorders (i.e. attention-deficit hyperactivity disorder or an eating disorder; N = 36), as reported by parents using the Health and Medical History Questionnaire. To account for misreporting bias, we also excluded adolescents with implausible scores for sweet propensity (N = 2). The final analysis group included 2,065 participants (Additional file 3).

### Statistical analyses

In the present study, we apply a causal analysis based on the potential outcomes framework which provides an approach to quantify causal effects based on hypothetical interventions. In this framework the causal effect for an individual is defined as the difference between the outcomes that would be observed for that individual with versus without the exposure or intervention under consideration. For the analyses to be valid, this framework requires meeting all causal identification assumptions, such as that there is a greater than zero probability of observing all values for the exposure in each covariate stratum (positivity assumption) [30].

Descriptive characteristics of the analysis group were stratified by sex with continuous variables displayed with mean and standard deviation (SD), and categorical variables with percentages. In the main analyses, psychosocial well-being, emotion-driven impulsiveness, sweet and fat propensity, country, sex, and BMI were obtained from W3; the remaining covariates were from W2 to account for the temporal order. W4 data on emotion-driven impulsiveness and sweet and fat propensity were used for sensitivity analyses. Missing values for any variable were imputed using single imputation via chained equations using the R-package ‘mice’ [31].

Addressing (Q1), we aimed to estimate the total (average) causal effects, as defined in terms of potential outcomes under hypothetical interventions [30], separately for psychosocial well-being and for emotion-driven impulsiveness on sweet and fat propensity. The average causal effect of interest is defined as the mean difference (MD) of sweet or fat propensity that would be observed in the same individuals under interventions increasing psychosocial well-being or decreasing emotion-driven impulsiveness. Thus, the MDs quantify the average causal effects if all adolescents had high (or moderate) levels of psychosocial well-being, or low (or moderate) levels of emotion-driven impulsiveness versus if all had low levels or high levels, respectively. The estimation was carried out by applying a semi-parametric doubly robust estimator: This estimator combines two models, an outcome regression model with a model for the exposure, such that only one of the two models needs to be correctly specified to obtain a consistent effect estimator [32]. In this study, the doubly robust estimator was obtained by Targeted Maximum Likelihood Estimation (TMLE) which uses the Super Learner to fit the two models in a data-driven way. The Super Learner is an algorithm that uses a statistical method (cross-validation) to estimate the performance of machine learning models. Its aim is to build the optimal weighted combination of predictions from a library of candidate machine learning algorithms. The machine learning algorithms impose minimal assumptions on the distribution of the data while yielding valid standard errors for statistical inference [16]. With this approach, we estimated four separate causal effects for the following four exposure and outcome pairs: Two effects with psychosocial well-being as categorical exposure and (i) sweet and (ii) fat propensity, each as continuous outcome variable. The ‘low’ exposure category was set as the reference category for psychosocial well-being. Analogously, two effects were estimated for emotion-driven impulsiveness as categorical exposure with (iii) sweet and (iv) fat propensity, each as continuous outcome. The ‘high’ exposure category was set as the reference category for emotion-driven impulsiveness. The estimation was carried out with the R-package ‘tmle3’ [33].

Addressing (Q2), we started by estimating (v) the causal effect of psychosocial well-being on emotion-driven impulsiveness (as continuous outcome) using the same TMLE approach as above. This forms part of a possibly mediated effect of psychosocial well-being via emotion-driven impulsiveness on sweet and fat propensity. Further, we estimated the direct and indirect causal effects of psychosocial well-being on (vi) sweet and (vii) fat propensity in a causal mediation analysis, a potential outcomes approach that extends linear structural equations to more general, e.g. non-linear, settings [17]. In terms of potential outcomes, the (average) direct effect is the effect of exposure (psychosocial well-being) while the mediator (emotion-driven impulsiveness) remains at the same natural value it would have under the reference category of exposure. The average causal mediated (i.e. indirect) effect, is the average difference in potential outcome under the natural values the mediator would take for the different exposure values, while keeping the actual exposure at its reference level along the direct path. The analysis relies on a set of flexible models for exposure, mediator, and outcome, which are then combined to obtain the direct and mediated effects using the mediational ‘g-formula’ [17, 34]. We performed the causal mediation analysis using the R-package ‘mediation’ [35]. An overview of variables that were obtained from respective waves for the analyses as well as detailed information on the statistical analyses can be found in the supplementary materials (Additional file 4).

We stratified the results by sex (male versus female) and BMI (thin or normal weight adolescents versus adolescents with overweight or obesity) since unhealthy food choices were shown to differ by sex [41] and BMI [42]. Sensitivity analyses were conducted to assess the robustness of our findings from our main analyses in which our exposure, mediator, and outcome variables were assessed within W3. Hence, one of our main sensitivity analyses accounted for the temporal order of our exposure measured at W3 and mediator and outcome variables at W4, respectively; moreover, assumptions underlying the causal analysis were checked where possible. A summary of different structural and modelling assumptions regarding temporality, alternative adjustment sets, potential positivity violations, and a triangulation approach using a parametric regression standardization can be found in the supplementary materials (Additional file 5). All analyses were carried out using the statistical software R (Version 4.1.3).

## Results

A total of 2,065 participants aged 10.1 to 16.2 years (mean = 13.4, SD = 0.8) with 53% female participants were included in this study (Table 1). At W3 male participants had an average score of 25.5 for sweet propensity and an average score of 25.9 for fat propensity; for female participants these were 24.5 and 24.2, respectively. The average score for emotion-driven impulsiveness for male and female participants was 24.1 and 25 points, respectively, while the well-being score was on average 37.8 points in the total analysis group. Corresponding characteristics of participants from the sensitivity analysis using W4 data (N = 855; mean age = 20.2, SD = 0.9) indicate that after seven years (in W4) participants had on average similar values than in the main analyses with the exception of a lower average score for sweet propensity (mean = 17.2, SD = 10.5) (Additional file 6).

**Table 1:** Descriptive characteristics of the analysis group

	Overall (N = 2,065)	Male (N = 970)	Female (N = 1,095)
*Continuous variables: mean (SD)*			
Sweet propensity, score (range: 0–68.4)	24.9 (11.2)	25.5 (11.0)	24.5 (11.3)
Fat propensity, score (range: 0–72.6)	25.0 (9.0)	25.9 (9.1)	24.2 (8.8)
Emotion-driven impulsiveness^a^, score (range: 12–48)	24.5 (7.5)	24.1 (7.5)	25.0 (7.5)
Psychosocial well-being^a^, score (range: 9–48)	37.8 (5.7)	38.6 (5.2)	37.1 (6.0)
Age, years (range: 10.1–16.2)	13.4 (0.8)	13.4 (0.8)	13.4 (0.7)
Media use, hours per week (range: 0–56; missing = 94)	19.5 (12.2)	23.1 (12.9)	16.3 (10.6)
*Categorical variables: %*			
Highest educational level of parents^b^			
high	48.2	47.5	48.8
low / medium	50.8	51.6	50.1
missing	1.0	0.8	1.1
Country			
Central Europe	37.8	39.4	36.3
Northern Europe	32.0	29.8	34.0
Southern Europe	30.2	30.8	29.7
BMI^c^			
Thin / normal weight	74.1	72.2	75.8
Overweight / obesity	25.7	27.8	24.2
Physical activity (sports club membership)			
no	62.1	66.5	58.3
yes	37.8	33.4	41.6
missing	0.1	0.1	0.1
Sleep quality			
0	22.9	26.7	19.5
1	39.9	40.8	39.0
2–3	33.1	28.5	37.2
missing	4.1	3.9	4.3

Information derived from W3 (2013–2014)

Percentages may not add up to 100% due to rounding

^a^ Displayed as continuous variable but included as categorical variables in the main analysis

^b^ Based on International Standard Classification of Education Maximum (ISCED; maximum of both parents)

^c^ Displayed as categorical variables but included as continuous variables in the main analysis

High psychosocial well-being, compared to low psychosocial well-being, decreased average sweet propensity [high: MD = -1.43, 95%-confidence interval (CI): -2.61 to -0.25]. A much less pronounced effect was estimated for average fat propensity (Table 2). Low levels of emotion-driven impulsiveness, compared to high levels, decreased average sweet propensity [low: MD = -2.07, CI: -3.26 to -0.87]. The positive effect also occurred for average fat propensity [low: MD = -1.85, CI: -2.88 to -0.81] (Table 2). When comparing estimates stratified by sex and BMI, the results did not differ (Sex: Additional file 7; BMI: Additional file 8).

Moderate or high versus low psychosocial well-being decreased average emotion-driven impulsiveness [moderate: MD = -2.53, CI: -3.36 to -1.71; high: MD = -4.95, CI: -5.79 to -4.12] (Table 2), Further, Table 3 shows the results from the mediation analysis, i.e. the direct and indirect (via emotion-driven impulsiveness) effects of psychosocial well-being on sweet and fat propensity. This suggests that the effect of high versus low psychosocial well-being on sweet [indirect effect: MD = -0.61, CI: -1.09 to -0.24] and fat [indirect effect: MD = -0.55, CI: -0.86 to -0.13] propensity is partly mediated by emotion-driven impulsiveness (Table 3). Note that, reassuringly, the total effects of psychosocial well-being on sweet and fat propensity, here, correspond to and confirm those in Table 2, despite being estimated under different modelling assumptions.

**Table 2:** Estimated effects of psychosocial well-being and emotion-driven impulsiveness on average fat and sweet propensity scores (N = 2,065 at W3; mean age = 13.4)

		Outcome [MD (95%-CI)]		
Exposure	Category levels	Emotion-driven impulsiveness	Sweet propensity	Fat propensity
Psychosocial well-being	Ref. level: low			
	moderate	-2.53 (-3.36, -1.71)	-0.30 (-1.44, 0.84)	-0.58 (-1.60, 0.45)
	high	-4.95 (-5.79, -4.12)	-1.43 (-2.61, -0.25)	-0.73 (-1.76, 0.30)
Emotion-driven impulsiveness	Ref. level: high			
	moderate	/	-1.01 (-2.16, 0.15)	-0.34 (-1.33, 0.66)
	low	/	-2.07 (-3.26, -0.87)	-1.85 (-2.88, -2.81)

W2: Variables measured in 2009–2010; W3: Variables measured in 2013–2014

Ref. level: Reference level; MD: Mean Difference; 95%-CI: 95% confidence interval

Exposure psychosocial well-being: adjusted for sweet or fat propensity (depending on outcome), psychosocial well-being, age, highest educational level of parents, physical activity, sleep quality, and media use at W2; sex, country, and BMI at W3

Exposure emotion-driven impulsiveness: adjusted for sweet or fat propensity (depending on outcome), psychosocial well-being, age, sex, highest educational level of parents, physical activity, sleep quality, and media use at W2; psychosocial well-being, sex, country, and BMI at W3

**Table 3:** Estimated direct, indirect, and total effects from the mediation analysis of psychosocial well-being (exposure) via emotion-driven impulsiveness (mediator) on fat and sweet propensity (N = 2,065 at W3, mean age = 13.4)

		Outcome [MD (95%-CI)]	
Category level of exposure psychosocial well-being; (Ref. level: low)		Sweet propensity	Fat propensity
moderate	Direct effect	-0.01 (-1.17, 1.14)	-0.60 (-1.62, 0.41)
	Indirect effect	-0.21 (-0.43, -0.01)	-0.21 (-0.44, -0.03)
	Total effect	-0.21 (-1.38, 0.94)	-0.81 (-1.84, 0.18)
	Prop. mediated	0.99 (-5.57, 5.17)*	0.26 (-1.32, 2.32)*
high	Direct effect	-0.85 (-2.09, 0.42)	-0.56 (-1.65, 0.52)
	Indirect effect	-0.61 (-1.09, -0.24)	-0.55 (-0.86, -0.13)
	Total effect	-1.45 (-2.66, -0.33)	-1.11 (-2.10, -0.01)
	Prop. mediated	0.42 (0.12, 1.86)*	0.50 (-0.04, 2.86)*

W2: Variables measured in 2009–2010; W3: Variables measured in 2013–2014

Ref. level: Reference level; MD: Mean Difference; 95%-CI: 95% confidence interval; Prop. mediated: Proportion mediated

†The proportion mediated provides an estimate of the extent to which the total effect is accounted for by the pathway through the mediating variable and is the ratio of the indirect effect on the total effect

*CI for proportion mediated contains values outside (0,1) when direct and indirect effects have potentially opposite signs

Mediator model for emotion-driven impulsiveness (W3; tertiles) adjusted for sweet or fat propensity score (depending on outcome), psychosocial well-being, age, sex, highest educational level of parents, country, physical activity, and sleep quality at W2; sex, country, and BMI at W3. Additional interaction terms between the exposure and covariates for the exposure-mediator and mediator-outcome relationships were included

Outcome models for sweet and fat propensity (W3; scores) adjusted for sweet or fat propensity score (depending on outcome), psychosocial well-being, age, sex, highest educational level of parents, country, sleep quality, and media use at W2; sex, country, and BMI at W3. Additional interaction terms between the exposure, mediator, and covariates for the exposure-outcome and mediator-outcome relationships were included

In a sensitivity analysis using mediator and outcome measurements at W4 instead of W3, i.e. 7 years after W3, we ensured that the exposure precedes the mediator and outcomes. For sweet propensity, we observed slightly increased estimates but signs remained the same; while for fat propensity, the signs were reversed (Additional file 9). We conducted further sensitivity analyses with alternative adjustment sets (physical activity, sleep quality, and media use measured at W3 instead of W2) since we cannot rule out that health-related variables may also be affected by psychosocial well-being and emotion-driven impulsiveness. Our results indicate that the estimated effects of both psychosocial well-being and emotion-driven impulsiveness on average sweet and fat propensity were attenuated (Additional file 10); however, they remained almost unchanged when adjusting for sociodemographic variables measured at W3 instead of W2 (Additional file 11). When identifying positivity violations, i.e. individuals with certain combinations of covariate values who were less likely to be in the exposed group, we conducted another sensitivity analysis without these positivity violations which confirmed the robustness of our results (Additional file 12). Using parametric regression standardization, which is a more standard statistical approach than TMLE, confirmed the findings in Tables 2 and 3 (Additional file 13). Results for all sensitivity analyses are available in the supplementary materials (Additional files 9–13).

## Discussion

To our knowledge, this is the first population-based study using a causal analysis to investigate and compare the effects of hypothetical interventions aiming at increasing psychosocial well-being or decreasing emotion-driven impulsiveness to reduce sweet and fat propensity in European adolescents. Our results highlight that an intervention on emotion-driven impulsiveness appears marginally more effective than on psychosocial well-being in reducing the consumption of sweet and fatty foods. In the following, it should be kept in mind that our results are somewhat difficult to compare with other studies due to differences in chosen populations, measurements, and statistical analyses. However, in order to interpret our results we identified studies conducted in adult populations investigating a similar relationship as in our study.

Addressing (Q1), our results suggest that if an intervention (on an adolescent) were to shift psychosocial well-being from a low to a high level, this would reduce the sweet propensity score by an average of 1.43 (CI: 0.25 to 2.61) points in adolescents. In view of the range and SD (Table 1) of the sweet propensity score, a change by about 1.43 points might be considered rather small. Additionally, our results provide some evidence that this effect is partly mediated by emotion-driven impulsiveness (addressing (Q2)). There is no clear evidence for an effect of the same intervention on fat propensity, though. In agreement with our findings, a previous study observed that a higher score on the Brief Resilience Scale, which assesses the perceived ability to bounce back or recover from stress, was associated with lower intake of sugar and confectionary, dairy desserts, and sugary fatty products [9]. Similar to our study there were no relevant effects for fats reported. Hence, our study complements the existing evidence with similar patterns observed in adolescents who have a greater demand for nutrients during their physical growth and brain development [36]. Higher psychosocial well-being may benefit cognitive control functions that facilitate healthy eating through goal setting, problem solving, and focusing on long-term goals [37].

In contrast, if a different intervention (on an adolescent) were to shift emotion-driven impulsiveness from a high to a low level, this would reduce the sweet propensity score by an average of 2.07 (CI: 0.87 to 3.26) points and the fat propensity score by an average of 1.85 (CI: 0.81 to 2.88) points. While the effect estimates are a little larger than for psychosocial well-being, they are still rather small in view of the range and SD (Table 1) of the sweet and fat propensity scores. Our results are consistent with a previous study in which a higher score on the Barratt Impulsiveness Scale, indicating higher levels of impulsivity, increased the average consumption of sugary and fatty foods by 2.66 g per day [15]. Another analysis based on the I.Family study population suggested that European adolescents with higher levels of emotion-driven impulsiveness were eating 0.25 more energy-dense snacks per day [14]. Different reasons may explain the effect of emotion-driven impulsiveness on sweet and fat propensity. First, ongoing brain maturation processes like neuronal myelination and synaptic pruning within the parietal cortex and prefrontal cortex cause deficits in impulse control and emotion regulation during adolescence [38]; hence, causing difficulties in resisting highly palatable food. Second, food taste was previously shown to be a stronger predictor of food choice than food healthiness in adolescents [39].

As part of (Q2) we estimated that higher psychosocial well-being decreased emotion-driven impulsiveness by an average of 4.95 points on the negative urgency scale (CI: 4.12 to 5.79) which complements previous findings on an inverse association between psychosocial well-being and emotion-driven impulsiveness [13, 40, 41]. Furthermore, our results suggest an indirect effect of psychosocial well-being on average sweet propensity (0.61, CI: 0.24 to 1.09) as well as fat propensity (0.55, CI: 0.13 to 0.86) via emotion-driven impulsiveness. Similarly, a previous study suggested an indirect effect of the Brief Resilience Scale on sugary fatty products via emotional eating, i.e. a reduction of sugary fatty products by 1.19 g per day [9]. Reward sensitivity appears to be dysregulated during stress exposure encouraging stress-related coping behaviors such as the consumption of highly palatable food [42].

Complex psychological constructs such as food choice are shaped by a multitude of proximal and distal factors [43]. Hence, our rather small effect sizes may be explained by the fact that psychosocial well-being and emotion-driven impulsiveness constitute only two psychological factors. Previous interventions targeting emotion-driven impulsiveness were usually conducted in clinical adult population samples and observed mainly small decreases in emotion-driven impulsiveness [44, 45]. Only one intervention study in an adolescent sample with overweight suggested a three-point decrease in emotion-driven impulsiveness after receiving cognitive behavioral therapy, structured physical activity, and dietary counseling. Decreasing emotion-driven impulsiveness resulted in lower BMI of moderate effect size [46]. Switching from the high to low emotion-driven impulsiveness category in our study, however, would require an average decrease in emotion-driven impulsiveness of at least 9 points; suggesting that the actual effect of an intervention in emotion-driven impulsiveness on sweet and fat propensity would be smaller than our estimated effects. Further, it is unclear (and in our study not empirically verifiable) whether an intervention on emotion-driven impulsiveness in adolescents with low psychosocial well-being might be successful. Hence, an intervention on psychosocial well-being could be more favorable, particularly when considering its indirect effect via emotion-driven impulsiveness and its more feasible interventions at the population level [47, 48]. Yet, actual interventions also yielded rather small effects on average due to heterogeneous settings such as different target populations or intervention intensities [49]. For instance, one intervention study on psychosocial well-being, also using the KINDL, found a small to moderate effect after a competence skill enhancement approach focusing on developing children’s positive emotions and strengths and a positive school climate [50]. Hence, switching from the low to high psychosocial well-being category would require an average increase in psychosocial well-being of at least 6 points.

Methods used to answer our research questions (Q1) and (Q2) rely on strong causal identifiability assumptions and have different advantages and disadvantages: Using a doubly robust method for (Q1) offers some degree of protection against model misspecifications, particularly those arising from an incorrect functional form such as linearity [51]. However, the data-adaptive algorithms require careful tuning for optimal performance [52]; details on our specific implementation of the Super Learner algorithm for TMLE can be found in the supplementary materials (Additional file 14). Applying a flexible method for causal mediation analysis for (Q2) provides additional information on the direct and indirect effect in the possible presence of exposure-mediator interactions [17]. These mediational effects may, however, be hard to interpret since the required assumptions are impossible to empirically verify and hard to justify [53].

This study was limited by the use of self-reported data and may be subject to measurement error. However, self-reported measures for our exposure and mediator variables are frequently used and have been validated [54]. Differential misreporting for our outcome variables is likely and also common in nutrition surveys, e.g. female participants and individuals with overweight or obesity tend to underreport their energy intake [55]. To tackle the possibility of reverse causation when measuring variables at the same time point, we conducted sensitivity analyses ensuring that the exposure preceded the mediator and outcome in time. These analyses yielded slightly increased estimates for sweet propensity and reversed estimates for fat propensity which are likely due to the long time span between examination periods that do not appropriately capture our expected cause and effect relationship over a short time period. Data on the mediator and outcomes were collected during the Covid-19 pandemic that may have affected study participants’ mental health and their ability to reflect on behaviors before the pandemic. Finally, we would need to meet all causal identification assumptions to be valid which are further discussed in the supplementary materials (Additional file 15). All of our limitations are, however, outweighed by important strengths of our analyses including confounding control, a flexible modelling approach, and various sensitivity analyses that confirm our results.

## Conclusion

Comparing the effects of psychosocial well-being and emotion-driven impulsiveness on sweet and fat propensity, our analyses suggest that interventions targeting emotion-driven impulsiveness might be marginally more effective to reduce sweet and fat propensity. Adolescents experiencing chronic stress such as low psychosocial well-being are prone to act more impulsively and are, hence, highly susceptible to the increased availability and promotion of these foods. This is alarming given the obesogenic environment in Western countries in which adolescents are surrounded by an abundance of these foods. Since adolescence is a time in which stress coping abilities are developed, our results specifically highlight that interventions targeting adaptive emotion regulation strategies might contribute to healthier eating. However, the feasibility and effectiveness of interventions aiming to decrease emotion-driven impulsiveness in adolescents in non-clinical settings needs to be further elucidated.

### Electronic supplementary material

Below is the link to the electronic supplementary material.

Additional file 1. List of food items in the sweet and fat propensity score

Additional file 2. Directed Acyclic Graph (DAG)

Additional file 3. Flow diagram of study participants

Additional file 4. Details on statistical analyses

Additional file 5. Details on sensitivity analyses

Additional file 6. Baseline characteristics of the study population from W3 and W4

Additional file 7. Estimated effects of psychosocial well-being and emotion-driven impulsiveness on average fat and sweet propensity; stratified by sex (at W3: N_male_: 970 and N_female_: 1,095)

Additional file 8. Estimated effects of psychosocial well-being and emotion-driven impulsiveness on average fat and sweet propensity; stratified by BMI (at W3: N_thin/normal weight_: 1,530 and N_overweight/obesity_: 535)

Additional file 9. Estimated effects of psychosocial well-being and emotion-driven impulsiveness on fat and sweet propensity with variables measured at W3/W4 (N = 855 at W4, mean age = 20.2)

Additional file 10. Estimated effects of psychosocial well-being and emotion-driven impulsiveness on fat and sweet propensity with health-related variables measured at W3 (N = 2,065 at W3)

Additional file 11. Estimated effects of psychosocial well-being and emotion-driven impulsiveness on fat and sweet propensity with sociodemographic variables measured at W3 (N = 2,065 at W3)

Additional file 12. Estimated effects of psychosocial well-being and emotion-driven impulsiveness on average fat and sweet propensity in subgroup without positivity violations (N = 1,954 at W3)

Additional file 13. Estimated effects of psychosocial well-being on average fat and sweet propensity using parametric regression standardisation (N = 2,065 at W3)

Additional file 14. Weights corresponding to each Super Learner algorithm obtained from the main analysis (Table 2) estimating the exposure or outcome mechanism

Additional file 15. Discussion on causal identification assumptions

Supplementary Material 16

## Data Availability

The datasets generated and/or analyzed during the current study are not publicly available but are available from the IDEFICS/I.Family consortia (http://www.ifamilystudy.eu/) on reasonable request.

## Abbreviations

CI: Confidence interval
DAG: Directed acyclic graph
FFQ: Food frequency questionnaire
IDEFICS: Identification and prevention of Dietary- and lifestyle-induced health EFfects In Children and infantS
ISCED: International Standard Classification of Education
MD: Mean difference
SD: Standard deviation
TMLE: Targeted maximum likelihood estimation
UPPS-P: Urgency, Premeditation, Perseverance, Sensation seeking, and Positive urgency

## References

[1] Birch LL, Doub AE (2014). Learning to eat: birth to age 2 y. Am J Clin Nutr.

[2] Sinha R (2018). Role of addiction and stress neurobiology on food intake and obesity. Biol Psychol.

[3] Adam TC, Epel ES (2007). Stress, eating and the reward system. Physiol Behav.

[4] Dallman MF, Pecoraro N, Akana SF, La Fleur SE, Gomez F, Houshyar H (2003). Chronic stress and obesity: a new view of comfort food. Proc Natl Acad Sci USA.

[5] Thanarajah SE, DiFeliceantonio AG, Albus K, Kuzmanovic B, Rigoux L, Iglesias S (2023). Habitual daily intake of a sweet and fatty snack modulates reward processing in humans. Cell Metabol.

[6] Park CL, Kubzansky LD, Chafouleas SM, Davidson RJ, Keltner D, Parsafar P (2023). Emotional well-being: what it is and why it matters. Affect Sci.

[7] Boehm JK. Positive psychological well-being and Cardiovascular Disease: exploring mechanistic and developmental pathways. Social and Personality Psychology Compass; 2021. 10.1111/spc3.12599 PMC9285725 35860033

[8] Boehm JK, Chen Y, Koga H, Mathur MB, Vie LL, Kubzansky LD (2018). Is optimism associated with healthier cardiovascular-related behavior? Meta-analyses of 3 health behaviors. Circ Res.

[9] Robert M, Shankland R, Bellicha A, Kesse-Guyot E, Deschasaux-Tanguy M, Andreeva VA (2022). Associations between resilience and food intake are mediated by emotional eating in the NutriNet-Santé Study. J Nutr.

[10] Burns R, Pachana NA (2015). Psychosocial Well-being. Encyclopedia of Geropsychology.

[11] Diener E, Napa Scollon C, Lucas RE, Diener E (2009). The Evolving Concept of Subjective Well-Being: the multifaceted nature of happiness. Assessing Well-Being: the Collected Works of Ed Diener.

[12] Cyders MA, Smith GT (2008). Emotion-based dispositions to rash action: positive and negative urgency. Psychol Bull.

[13] Do S, Coumans JM, Börnhorst C, Pohlabeln H, Reisch LA, Danner UN (2022). Associations between psychosocial well-being, stressful life events and emotion-driven impulsiveness in European adolescents. J Youth Adolesc.

[14] Coumans JM, Danner UN, Intemann T, De Decker A, Hadjigeorgiou C, Hunsberger M (2018). Emotion-driven impulsiveness and snack food consumption of European adolescents: results from the I. Family study. Appetite.

[15] Bénard M, Bellisle F, Kesse-Guyot E, Julia C, Andreeva VA, Etilé F (2019). Impulsivity is associated with food intake, snacking, and eating disorders in a general population. Am J Clin Nutr.

[16] Van der Laan MJ, Rose S. Targeted learning: causal inference for observational and experimental data. Springer; 2011.

[17] VanderWeele T. Explanation in causal inference: methods for mediation and interaction. Oxford University Press; 2015.

[18] Ahrens W, Siani A, Adan R, De Henauw S, Eiben G, Gwozdz W (2017). Cohort Profile: the transition from childhood to adolescence in European children–how I. Family extends the IDEFICS cohort. Int J Epidemiol.

[19] Pala V, Reisch LA, Lissner L. Dietary behaviour in children, adolescents and families: the eating habits questionnaire (EHQ). Instruments for health surveys in children and adolescents. Springer; 2019. pp. 103–33.

[20] Huybrechts I, Börnhorst C, Pala V, Moreno L, Barba G, Lissner L (2011). Evaluation of the children’s eating habits Questionnaire used in the IDEFICS study by relating urinary calcium and potassium to milk consumption frequencies among European children. Int J Obes.

[21] Lanfer A, Hebestreit A, Ahrens W, Krogh V, Sieri S, Lissner L (2011). Reproducibility of food consumption frequencies derived from the children’s eating habits Questionnaire used in the IDEFICS study. Int J Obes.

[22] Whiteside SP, Lynam DR (2001). The five factor model and impulsivity: using a structural model of personality to understand impulsivity. Pers Indiv Differ.

[23] Whiteside SP, Lynam DR, Miller JD, Reynolds SK (2005). Validation of the UPPS impulsive behaviour scale: a four-factor model of impulsivity. Eur J Pers.

[24] Ravens-Sieberer U, Bullinger M. KINDL-R. Questionnaire for measuring health-related quality of life in children and adolescents, revised version. 2000 [Available from: https://www.kindl.org/english/publications/.

[25] Bullinger M, Brütt AL, Erhart M, Ravens-Sieberer U (2008). Psychometric properties of the KINDL-R questionnaire: results of the BELLA study. Eur Child Adolesc Psychiatry.

[26] Stok FM, Hoffmann S, Volkert D, Boeing H, Ensenauer R, Stelmach-Mardas M (2017). The DONE framework: creation, evaluation, and updating of an interdisciplinary, dynamic framework 2.0 of determinants of nutrition and eating. PLoS ONE.

[27] UNESCO Institute for Statistics (2012). International Standard Classification and Education ISCED 2011.

[28] Cole TJ, Lobstein T (2012). Extended international (IOTF) body mass index cut-offs for thinness, overweight and obesity. Pediatr Obes.

[29] Sprengeler O, Wirsik N, Hebestreit A, Herrmann D, Ahrens W (2017). Domain-specific self-reported and objectively measured physical activity in children. Int J Environ Res Public Health.

[30] Hernán M, Robins J (2020). Causal inference: what If.: Boca.

[31] van Buuren S, Groothuis-Oudshoorn K (2011). Mice: multivariate imputation by chained equations in R. J Stat Softw.

[32] Robins JM, Rotnitzky A, Zhao LP (1994). Estimation of regression coefficients when some regressors are not always observed. J Am Stat Assoc.

[33] Coyle J. tmle3: The Extensible TMLE Framework 2021 [R package version 0.2.0]. Available from: https://github.com/tlverse/tmle3.

[34] Imai K, Keele L, Tingley D, Yamamoto T. Causal mediation analysis using R. advances in social science research using R. Springer; 2010. pp. 129–54.

[35] Tingley D, Yamamoto T, Hirose K, Keele L, Imai K (2014). Mediation: R Package for Causal Mediation Analysis. J Stat Softw.

[36] Spear BA (2002). Adolescent growth and development. J Am Diet Assoc.

[37] DeSteno D (2009). Social emotions and intertemporal choice: hot mechanisms for building social and economic capital. Curr Dir Psychol Sci.

[38] Defoe IN, Dubas JS, Figner B, Van Aken MA (2015). A meta-analysis on age differences in risky decision making: adolescents versus children and adults. Psychol Bull.

[39] van Meer F, van der Laan LN, Eiben G, Lissner L, Wolters M, Rach S (2019). Development and body mass inversely affect children’s brain activation in dorsolateral prefrontal cortex during food choice. NeuroImage.

[40] Ravert RD, Donnellan MB (2021). Impulsivity and sensation seeking: differing associations with psychological well-being. Appl Res Qual Life.

[41] Rose MH, Nadler EP, Mackey ER (2018). Impulse control in negative mood states, emotional eating, and food addiction are associated with lower quality of life in adolescents with severe obesity. J Pediatr Psychol.

[42] Baik JH (2020). Stress and the dopaminergic reward system. Exp Mol Med.

[43] Götz FM, Gosling SD, Rentfrow PJ (2022). Small effects: the indispensable foundation for a cumulative psychological science. Perspect Psychol Sci.

[44] Preuss H, Pinnow M, Schnicker K, Legenbauer T (2017). Improving inhibitory control abilities (ImpulsE)—A promising approach to treat impulsive eating?. Eur Eat Disorders Rev.

[45] Garcia-Caballero A, Torrens-Lluch M, Ramírez-Gendrau I, Garrido G, Vallès V, Aragay N (2018). The efficacy of motivational intervention and cognitive-behavioral therapy for pathological gambling. Adicciones.

[46] Delgado-Rico E, Río-Valle JS, Albein-Urios N, Caracuel A, González-Jiménez E, Piqueras MJ (2012). Effects of a multicomponent behavioral intervention on impulsivity and cognitive deficits in adolescents with excess weight. Behav Pharmacol.

[47] Froh JJ, Sefick WJ, Emmons RA (2008). Counting blessings in early adolescents: an experimental study of gratitude and subjective well-being. J Sch Psychol.

[48] Vinci C, Peltier M, Waldo K, Kinsaul J, Shah S, Coffey SF (2016). Examination of trait impulsivity on the response to a brief mindfulness intervention among college student drinkers. Psychiatry Res.

[49] Kubzansky LD, Kim ES, Boehm JK, Davidson RJ, Huffman JC, Loucks EB (2023). Interventions to modify psychological well-being: Progress, promises, and an agenda for future research. Affect Sci.

[50] Elfrink TR, Goldberg JM, Schreurs KM, Bohlmeijer ET, Clarke AM (2017). Positive educative programme: a whole school approach to supporting children’s well-being and creating a positive school climate: a pilot study. Health Educ.

[51] Keil AP, Mooney SJ, Jonsson Funk M, Cole SR, Edwards JK, Westreich D (2018). Resolving an apparent paradox in doubly robust estimators. Am J Epidemiol.

[52] Naimi AI, Balzer LB (2018). Stacked generalization: an introduction to super learning. Eur J Epidemiol.

[53] Andrews RM, Didelez V (2020). Insights into the cross-world independence assumption of causal mediation analysis. Epidemiology.

[54] Sandvik E, Diener E, Seidlitz L. Subjective well-being: The convergence and stability of self-report and non-self-report measures. Assessing well-being: The collected works of Ed Diener. 2009:119 – 38.

[55] Sichert-Hellert W, Kersting M, Schöch G (1998). Underreporting of energy intake in 1 to 18 year old German children and adolescents. Z für Ernährungswissenschaft.

