# Supplementary material for: The role of psychosocial well-being and emotion-driven impulsiveness in food choices of European adolescents
Source: Int J Behav Nutr Phys Act. 2024 Jan 2;21:1. doi: 10.1186/s12966-023-01551-w (PMC10759484; doi:10.1186/s12966-023-01551-w)
Supplement: Supplementary file 1 — Additional file 1. List of food items in the sweet and fat propensity score [file 12966_2023_1551_MOESM1_ESM.docx]

**Additional file 1. List of food items in the sweet and fat propensity score**

|  | **Sweet propensity** | **Fat propensity** |
| --- | --- | --- |
| Vegetables | / | - Fried potatoes |
| Fresh fruits | - Fresh fruits (also as freshly squeezed juice) with added sugar | / |
| Drinks | - Fruit juices (100% fruit), packaged (orange juice, apple juice, local examples, etc.) - Diet carbonated drinks (e.g. diet cola, local examples, etc.) - Sugar sweetened drinks, not carbonated (e.g. bottled ice tea, syrup-based drinks and similar, fruit juices with less than 100% fruit, sports drinks, non-alcoholic wine, local examples, etc.) |  |
| Breakfast cereals | - Sweetened or sugar added breakfast cereals and sweetened crisp muesli (e.g. local examples, etc.) | / |
| Milk | - Sweetened and/or flavoured milk (e.g. chocolate powder, addition of sugar, honey, local examples, etc.) | - Plain unsweetened milk - Whole (full fat) milk |
| Yoghourt | - Sweetened and/or flavoured yoghourt (e.g. Actimel®, LC1®, local examples, etc.) | - Plain unsweetened yoghourt - Whole (full fat) yoghourt |
| Fish | / | - Fish, fried and/or coated (e.g. fish fingers, local examples) |
| Meat and meat products | / | - Cold cuts and preserved, ready to cook meat product (e.g. local examples) - Fried meat (beef, pork, local examples) |
| Eggs and mayonnaise | / | - Fried or scrambled eggs - Mayonnaise and mayonnaise based products (e.g. local examples) |
| Cheese | / | - Sliced cheese (e.g. local examples) - Spreadable cheese (e.g. local examples) - Grated cheese |
| Spreadable products | - Jam, honey - Chocolate, nut-based spread | - Chocolate or nut-based spread - Butter, margarine on bread |
| Snacks | - Snacks like chocolate, candy bars (Mars, Lion, Kit Kat, local examples, etc.) - Snacks like candies, loose candies, marshmallow (e.g. local examples, etc.) - Snacks like biscuits, packaged cakes, or pastries and puddings (e.g. like sweet bakery products, local examples, etc.) - Ice cream, milk or fruit based bars (local examples, etc.) | - Nuts and seeds (e.g. local examples, etc.) - Snacks like crisps, corn crisps, popcorn, etc. (e.g. local examples, etc.) - Snacks like savoury pastries and fritters (e.g. cheese pie, sausage pie, pancakes, local examples, etc.) - Snacks like chocolate, candy bars (Mars, Lion, Kit Kat, local examples, etc.) - Snacks like biscuits, packaged cakes, or pastries and puddings (e.g. like sweet bakery products, local examples, etc.) - Ice cream, milk or fruit based bars (local examples, etc.) |
