# Supplementary material for: The role of psychosocial well-being and emotion-driven impulsiveness in food choices of European adolescents
Source: Int J Behav Nutr Phys Act. 2024 Jan 2;21:1. doi: 10.1186/s12966-023-01551-w (PMC10759484; doi:10.1186/s12966-023-01551-w)
Supplement: Supplementary file 2 — Additional file 2. Directed Acyclic Graph (DAG) [file 12966_2023_1551_MOESM2_ESM.docx]

**Additional file 2. Directed Acyclic Graph (DAG)**

The following steps were undertaken to construct the DAG:

1. Check influences on diet within the Determinants Of Nutrition and Eating (DONE) Framework^^[[1]](#footnote-1)^^
   1. Apply filter to population of interest: children, school-aged children
   2. Apply filter to level of interest: interpersonal, individual level
   3. Reduction of determinants: based on prioritization rounds in PEN project^^[[2]](#footnote-2)^^
   4. Reconsideration of non-prioritized determinants based on relationship strength as displayed in the DONE Framework Web tool^^[[3]](#footnote-3)^^
   5. Exclude determinants that are very specific to diet (e.g. food beliefs, habits, etc.)
2. Check determinants for emotion-driven impulsiveness via literature search
3. Check determinants for psychosocial well-being if associated with either food choices, emotion-driven impulsiveness or mediator-outcome confounding variable

4) Check relationships between confounding variables

This DAG was created with DAGitty, a browser based application that enables drawing causal diagrams^[[4]](#footnote-4)^.


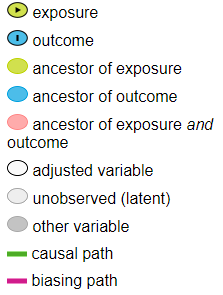


Legend


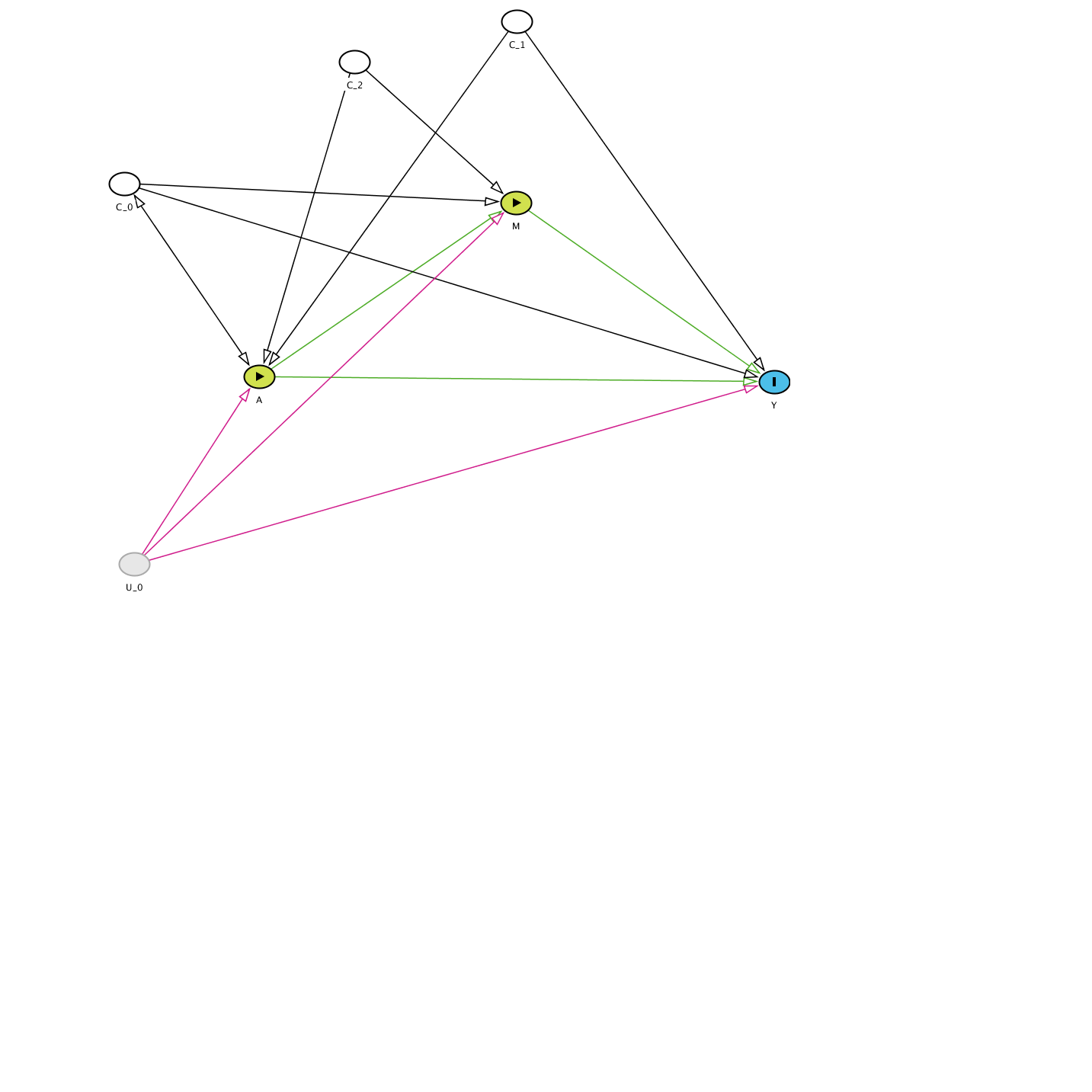


Abbreviations*


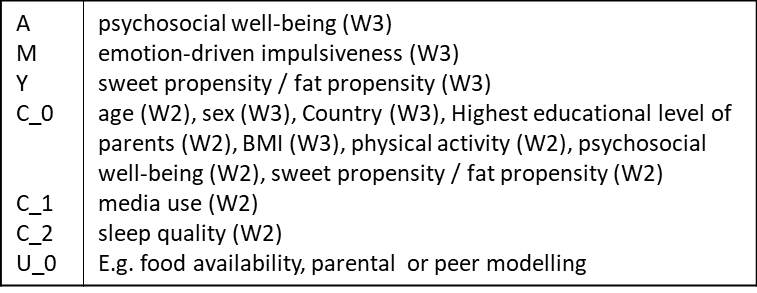


*All covariates (C_0, C_1, C_2) measured at W2 were used in the main analyses except of sex and country (time-fixed, i.e. unlikely to change over time) as well as BMI (assumed to affect psychosocial well-being over a short time period).

1. Stok FM, Hoffmann S, Volkert D, Boeing H, Ensenauer R, Stelmach-Mardas M, et al. The DONE framework: Creation, evaluation, and updating of an interdisciplinary, dynamic framework 2.0 of determinants of nutrition and eating. PLOS ONE. 2017;12(2):e0171077. [↑](#footnote-ref-1)
2. Garnica Rosas L, Mensink GBM, Finger JD, Schienkiewitz A, Do S, Wolters M, et al. Selection of key indicators for European policy monitoring and surveillance for dietary behaviour, physical activity and sedentary behaviour. International Journal of Behavioral Nutrition and Physical Activity. 2021;18(1):48. [↑](#footnote-ref-2)
3. [view interactive data • DONE Universität Konstanz (uni-konstanz.de)](https://www.uni-konstanz.de/DONE/view-interactive-data/) (Last accessed on 9 June 2023) [↑](#footnote-ref-3)
4. Textor J, Van der Zander B, Gilthorpe MS, Liśkiewicz M, Ellison GT. Robust causal inference using directed acyclic graphs: the R package ‘dagitty’. International Journal of Epidemiology. 2016;45(6):1887-94. [↑](#footnote-ref-4)
