# Supplementary figures and images for: The role of psychosocial well-being and emotion-driven impulsiveness in food choices of European adolescents
Source: Int J Behav Nutr Phys Act. 2024 Jan 2;21:1. doi: 10.1186/s12966-023-01551-w (PMC10759484; doi:10.1186/s12966-023-01551-w)

**Additional file 3. Flow diagram of study participants**


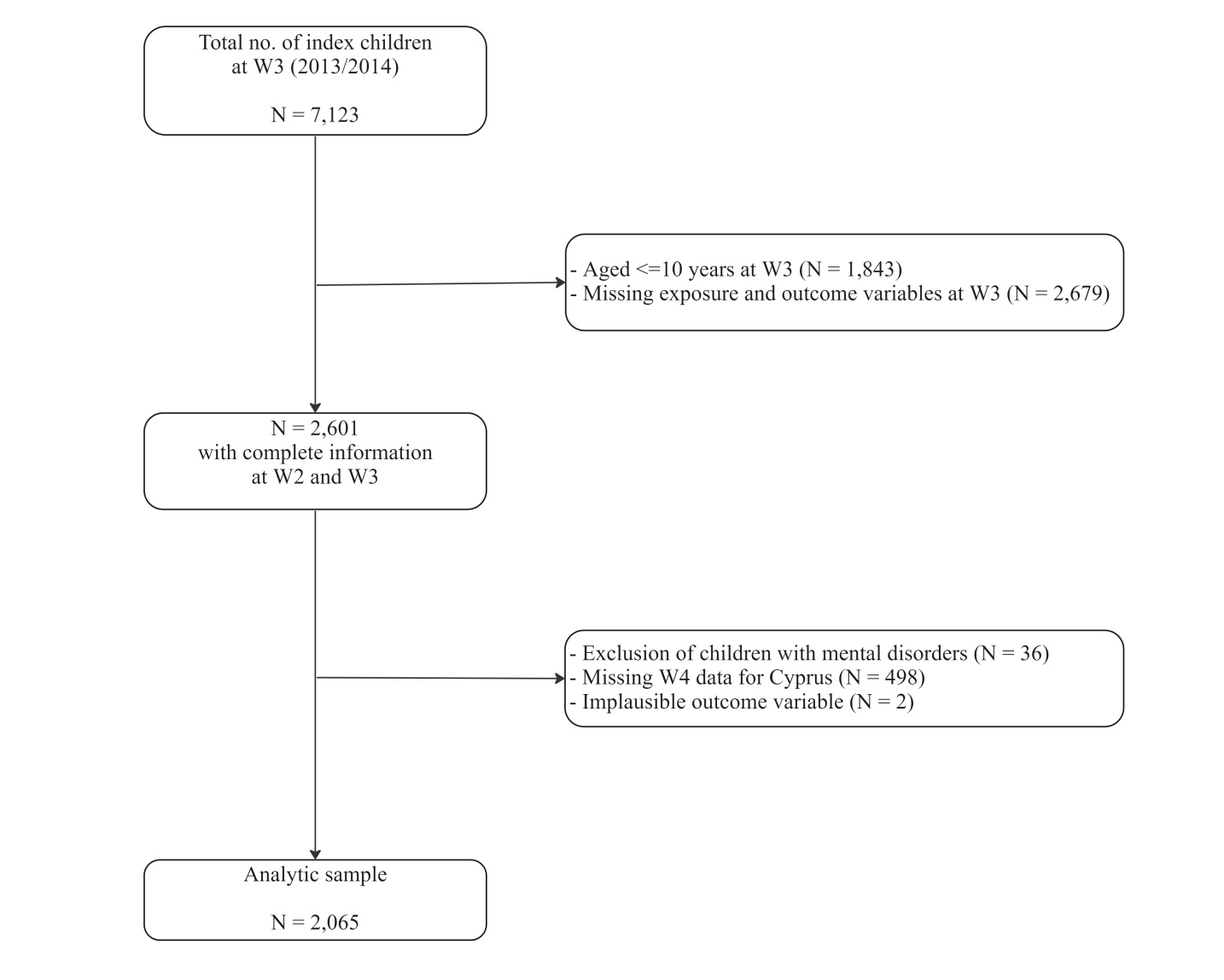

Supplement: Supplementary file 3 — Additional file 3. Flow diagram of study participants [file 12966_2023_1551_MOESM3_ESM.docx]
