# Supplementary material for: The role of psychosocial well-being and emotion-driven impulsiveness in food choices of European adolescents
Source: Int J Behav Nutr Phys Act. 2024 Jan 2;21:1. doi: 10.1186/s12966-023-01551-w (PMC10759484; doi:10.1186/s12966-023-01551-w)
Supplement: Supplementary file 4 — Additional file 4. Details on statistical analyses [file 12966_2023_1551_MOESM4_ESM.docx]

**Additional file 4. Details on statistical analyses**

Missing data

Multiple imputation (MI) using Fully Conditional Specification (FCS) is a statistical approach that enables an imputation model for each variable and the inclusion of both continuous and categorical variables in the imputation model (4). To account for missing data in our covariates (0.1 to 4.1%), we applied MI using FCS that was implemented by the MICE algorithm. The MICE algorithm produces many complete copies of a dataset, each with different imputations of the missing data. We used one iteration for our imputation models.

Targeted maximum likelihood estimation (TMLE)

TMLE is a doubly robust substitution-based estimation strategy (5). It allows two chances for confounding adjustment by fitting and combining two models to estimate the effect of interest (6, 7): The ‘outcome’ model in which the outcome is conditional on the exposure and all covariates; and the ‘exposure’ model in which the exposure is conditional on all covariates.

In TMLE, the exposure and outcome model are predicted using the cross-validated targeted maximum likelihood estimator (the ‘tmle3’ package (8)), including the cross-validated ensemble Super Learner which is a general loss-based learning method designed to find the optimal combination of a set of learners (9). Each ensemble learner was fitted using ten-fold cross validation. We adapted a pre-specified library of ensemble learners from the workshop ‘Introduction to Machine Learning and Causal Inference’ given by Prof. Dr. Ashley Naimi as part of the ‘4th RUHR School of Modern Epidemiology’ in 2022 (Material available at <https://github.com/ainaimi/mlci_shortcourse>). The library included the following ensemble learners for our exposure and outcome model with default tuning parameters:

| Exposure model | Outcome model |
| --- | --- |
| - Simple mean - Random forests - Generalized linear models with elastic net regularization - Extreme gradient boosting - Support vector machines | - Simple mean - Generalized linear models - Random forests - Generalized linear models with elastic net regularization - Extreme gradient boosting - Multivariate adaptive regression splines - Support vector machines |

Causal mediation analysis

Causal mediation analysis extends linear structural equations to non-linear settings and further allows the inclusion of a exposure-mediator interaction (10).We chose a simulation-based Monte-Carlo approach (the ‘mediation’ package (11)) which allows for more flexible modeling of mediator and outcome models than the regression-based method (12). In our analyses, an ordered logistic regression was used for the mediator model; and a linear regression for the outcome model. Besides allowing for an exposure-mediator interaction, we further included interaction terms such as second-order polynomials and two-way interactions between the main terms since we assumed non-linear relationships between our variables. A complete list of interaction terms can be found in the table below (‘Interaction terms used in causal mediation analyses’). Confidence intervals were estimated via the bootstrap (default: basic percentile intervals) with 10,000 resamples.

Model specification

A complete list of exposure, mediator, outcome and confounder variables and their time of measurement for each of our models are listed below:

Models (i) - (v): TMLE

Models (vi) and (vii): Causal mediation analysis

| **Model** | **Variables (Wave)** | **Scale** | **Outcome** | **Mediator** | **Exposure** | **Confounder** | | |
| --- | --- | --- | --- | --- | --- | --- | --- | --- |
|  |  |  |  |  |  | Exposure-Outcome | Exposure-Mediator | Mediator-Outcome |
| (i) | Sweet propensity (W3) | continuous | X |  |  |  |  |  |
|  | Psychosocial well-being (W3) | categorical |  |  | X |  |  |  |
|  | Sweet propensity (W2) | continuous |  |  |  | X |  |  |
|  | Psychosocial well-being (W2) | continuous |  |  |  | X |  |  |
|  | Age (W2) | continuous |  |  |  | X |  |  |
|  | Sex (W2) | binary |  |  |  | X |  |  |
|  | Country (W2) | categorical |  |  |  | X |  |  |
|  | Highest educational level of parents (W2) | binary |  |  |  | X |  |  |
|  | BMI (W3) | continuous |  |  |  | X |  |  |
|  | Physical activity (W2) | binary |  |  |  | X |  |  |
|  | Media use (W2) | continuous |  |  |  | X |  |  |
| (ii) | Fat propensity (W3) | continuous | X |  |  |  |  |  |
|  | Psychosocial well-being (W3) | categorical |  |  | X |  |  |  |
|  | Fat propensity (W2) | continuous |  |  |  | X |  |  |
|  | Psychosocial well-being (W2) | continuous |  |  |  | X |  |  |
|  | Age (W2) | continuous |  |  |  | X |  |  |
|  | Sex (W2) | binary |  |  |  | X |  |  |
|  | Country (W2) | categorical |  |  |  | X |  |  |
|  | Highest educational level of parents (W2) | binary |  |  |  | X |  |  |
|  | BMI (W3) | continuous |  |  |  | X |  |  |
|  | Physical activity (W2) | binary |  |  |  | X |  |  |
|  | Media use (W2) | continuous |  |  |  | X |  |  |
| (iii) | Sweet propensity (W3) | continuous | X |  |  |  |  |  |
|  | Emotion-driven impulsiveness (W3) | categorical |  |  | X |  |  |  |
|  | Sweet propensity (W2) | continuous |  |  |  | X |  |  |
|  | Psychosocial well-being (W3) | categorical |  |  |  | X |  |  |
|  | Psychosocial well-being (W2) | continuous |  |  |  | X |  |  |
|  | Age (W2) | continuous |  |  |  | X |  |  |
|  | Sex (W2) | binary |  |  |  | X |  |  |
|  | Country (W2) | categorical |  |  |  | X |  |  |
|  | Highest educational level of parents (W2) | binary |  |  |  | X |  |  |
|  | BMI (W3) | continuous |  |  |  | X |  |  |
|  | Physical activity (W2) | binary |  |  |  | X |  |  |
|  | Sleep quality (W2) | continuous |  |  |  | X |  |  |
| (iv) | Fat propensity (W3) | continuous | X |  |  |  |  |  |
|  | Emotion-driven impulsiveness (W3) | categorical |  |  | X |  |  |  |
|  | Fat propensity (W2) | continuous |  |  |  | X |  |  |
|  | Psychosocial well-being (W3) | categorical |  |  |  | X |  |  |
|  | Psychosocial well-being (W2) | continuous |  |  |  | X |  |  |
|  | Age (W2) | continuous |  |  |  | X |  |  |
|  | Sex (W2) | binary |  |  |  | X |  |  |
|  | Country (W2) | categorical |  |  |  | X |  |  |
|  | Highest educational level of parents (W2) | binary |  |  |  | X |  |  |
|  | BMI (W3) | continuous |  |  |  | X |  |  |
|  | Physical activity (W2) | binary |  |  |  | X |  |  |
|  | Sleep quality (W2) | continuous |  |  |  | X |  |  |
| (v) | Emotion-driven impulsiveness (W3) | continuous | X |  |  |  |  |  |
|  | Psychosocial well-being (W3) | categorical |  |  | X |  |  |  |
|  | Psychosocial well-being (W2) | continuous |  |  |  | X |  |  |
|  | Age (W2) | continuous |  |  |  | X |  |  |
|  | Sex (W2) | binary |  |  |  | X |  |  |
|  | Country (W2) | categorical |  |  |  | X |  |  |
|  | Highest educational level of parents (W2) | binary |  |  |  | X |  |  |
|  | BMI (W3) | continuous |  |  |  | X |  |  |
|  | Physical activity (W2) | binary |  |  |  | X |  |  |
|  | Sleep quality (W2) | continuous |  |  |  | X |  |  |
| (vi) | Emotion-driven impulsiveness (W3) | categorical | X |  |  |  |  |  |
| mediator model | Psychosocial well-being (W3) | categorical |  |  | X |  |  |  |
|  | Psychosocial well-being (W2) | continuous |  |  |  | X |  |  |
|  | Age (W2) | continuous |  |  |  | X | X | X |
|  | Sex (W2) | binary |  |  |  | X | X | X |
|  | Country (W2) | categorical |  |  |  | X | X | X |
|  | Highest educational level of parents (W2) | binary |  |  |  | X | X | X |
|  | BMI (W3) | continuous |  |  |  | X | X | X |
|  | Physical activity (W2) | binary |  |  |  | X | X | X |
|  | Sleep quality (W2) | continuous |  |  |  |  | X |  |
| (vi) | Sweet propensity (W3) | continuous | X |  |  |  |  |  |
| outcome model | Emotion-driven impulsiveness (W3) | continuous |  | X |  |  |  |  |
|  | Psychosocial well-being (W3) | categorical |  |  | X |  |  |  |
|  | Sweet propensity (W2) | continuous |  |  |  | X |  |  |
|  | Psychosocial well-being (W2) | continuous |  |  |  | X |  |  |
|  | Age (W2) | continuous |  |  |  | X | X | X |
|  | Sex (W2) | binary |  |  |  | X | X | X |
|  | Country (W2) | categorical |  |  |  | X | X | X |
|  | Highest educational level of parents (W2) | binary |  |  |  | X | X | X |
|  | BMI (W3) | continuous |  |  |  | X | X | X |
|  | Physical activity (W2) | binary |  |  |  | X | X | X |
|  | Media use (W2) | continuous |  |  |  | X |  |  |
| (vii) | Emotion-driven impulsiveness (W3) | categorical | X |  |  |  |  |  |
| mediator model | Psychosocial well-being (W3) | categorical |  |  | X |  |  |  |
|  | Psychosocial well-being (W2) | continuous |  |  |  | X |  |  |
|  | Age (W2) | continuous |  |  |  | X | X | X |
|  | Sex (W2) | binary |  |  |  | X | X | X |
|  | Highest educational level of parents (W2) | binary |  |  |  | X | X | X |
|  | Country (W2) | categorical |  |  |  | X | X | X |
|  | BMI (W3) | continuous |  |  |  | X | X | X |
|  | Physical activity (W2) | binary |  |  |  | X | X | X |
|  | Sleep quality (W2) | continuous |  |  |  |  | X |  |
| (vii) | Fat propensity (W3) | continuous | X |  |  |  |  |  |
| outcome model | Emotion-driven impulsiveness (W3) | categorical |  | X |  |  |  |  |
|  | Psychosocial well-being (W3) | categorical |  |  | X |  |  |  |
|  | Fat propensity (W2) | continuous |  |  |  | X |  |  |
|  | Psychosocial well-being (W2) | continuous |  |  |  | X |  |  |
|  | Age (W2) | continuous |  |  |  | X | X | X |
|  | Sex (W2) | binary |  |  |  | X | X | X |
|  | Country (W2) | categorical |  |  |  | X | X | X |
|  | Highest educational level of parents (W2) | binary |  |  |  | X | X | X |
|  | BMI (W3) | continuous |  |  |  | X | X | X |
|  | Physical activity (W2) | binary |  |  |  | X | X | X |
|  | Media use (W2) | continuous |  |  |  | X |  |  |

Interaction terms used in causal mediation analyses

| **Variables (Wave)** | **Interaction terms** | | | | | | | | | | | |
| --- | --- | --- | --- | --- | --- | --- | --- | --- | --- | --- | --- | --- |
|  | EDI (W3) | PWB (W3) | PWB (W2) | Sweet (6) or fat (7) propensity (W2) | Age (W2) | Sex (W2) | Country (W3) | ISCED (W2) | BMI (W3) | PA (W2) | Sleep (W2) | Media use (W2) |
| **Model (vi) or (vii) (mediator model)** | | | | | | | | | | | | |
| PWB (W3) |  |  |  |  | X | X | X | X | X |  |  |  |
| PWB (W2) |  |  |  |  | X | X | X | X |  | X | X |  |
| Age (W2) |  | X | X |  | X | X |  |  |  | X | X |  |
| Sex (W2) |  | X | X |  | X |  |  |  | X | X | X |  |
| Country (W3) |  | X | X |  |  |  |  | X |  |  |  |  |
| ISCED (W2) |  | X | X |  |  |  | X |  |  |  |  |  |
| BMI (W3) |  | X |  |  |  | X |  |  | X |  |  |  |
| PA (W2) |  |  | X |  | X | X |  |  |  |  |  |  |
| Sleep (W2) |  |  | X |  | X | X |  |  |  |  |  |  |
| **Model (vi) or (vii) (outcome model)** | | | | | | | | | | | | |
| EDI (W3) |  | X |  |  | X | X | X | X | X |  |  |  |
| PWB (W3) | X |  |  |  | X | X | X | X | X |  |  |  |
| Sweet (6) or fat (7) propensity (W2) |  |  | X | X | X | X |  | X |  |  |  |  |
| PWB (W2) |  |  | X | X | X | X | X | X |  | X |  | X |
| Age (W2) | X | X | X | X | X | X |  |  | X | X |  | X |
| Sex (W2) | X | X | X | X | X |  |  |  | X | X |  | X |
| Country (W3) | X | X | X |  |  |  |  | X |  |  |  |  |
| ISCED (W2) | X | X | X | X |  |  | X |  |  |  |  |  |
| BMI (W3) | X | X |  |  | X | X |  |  | X |  |  |  |
| PA (W2) |  |  | X |  | X | X |  |  |  |  |  |  |
| Media use (W2) |  |  | X |  | X | X |  |  |  |  |  | X |
| Abbreviations:  Psychosocial well-being (PWB), Emotion-driven impulsiveness (EDI),  Highest educational level of parents (ISCED), Physical activity (PA), Sleep quality (Sleep) | | | | | | | | | | | | |
