# Supplementary material for: The role of psychosocial well-being and emotion-driven impulsiveness in food choices of European adolescents
Source: Int J Behav Nutr Phys Act. 2024 Jan 2;21:1. doi: 10.1186/s12966-023-01551-w (PMC10759484; doi:10.1186/s12966-023-01551-w)
Supplement: Supplementary file 5 — Additional file 5. Details on sensitivity analyses [file 12966_2023_1551_MOESM5_ESM.docx]

**Additional file 5. Details on sensitivity analyses**

In addition to our main analyses (**Table 2**), we conducted the following sensitivity analyses checking the following assumptions:

1. Temporality was addressed in an analysis ensuring exposures precede the mediator and outcome by including the exposure from W3 in the model together with mediator and outcomes from W4 (as opposed to exposures, mediators and outcomes from W3 in the main model). The sufficient adjustment set of confounders was derived from W2 and W3 (**Additional file 9**).
2. We conducted additional analyses with alternative adjustment sets with:

a) health-related confounders derived from W3 instead of W2 that are assumed to be associated with psychosocial well-being, i.e., physical activity, sleep quality, and media use, (**Additional file 10**) and

b) sociodemographic confounders derived from W3 instead of W2, since a change in age and parental educational level may influence the investigated relationships in a different way (**Additional file 11**).

1. One of the causal identification assumptions that can be empirically verified is the positivity assumption. It requires exposed and unexposed individuals need to be within all confounding levels. Hence, we further conducted a sensitivity analysis that evaluated potential violations with propensity score overlap plots. We identified a lack of overlap for several confounders and, therefore, restricted the sample to study participants with (variables shown are scaled at the mean; **Additional file 12**):

- Psychosocial well-being (W2) >= -3,
- Age (W2) >= 8,
- Media use (W2) <= 3,
- Sweet propensity (W2) <= 2.5, and
- Fat propensity (W2) <= 2.5

1. An additional analysis was conducted using parametric regression standardisation, also known as g-formula, which standardises the mean outcome to the confounder distribution (13). The estimation was carried out with the R-package ‘stdReg’ (14). Similar to the causal mediation analyses, we included in our linear regression models second-order polynomials and two-way interactions between the main terms (**Additional file 13**).
