# Supplementary material for: The role of psychosocial well-being and emotion-driven impulsiveness in food choices of European adolescents
Source: Int J Behav Nutr Phys Act. 2024 Jan 2;21:1. doi: 10.1186/s12966-023-01551-w (PMC10759484; doi:10.1186/s12966-023-01551-w)
Supplement: Supplementary file 6 — Additional file 6. Baseline characteristics of the study population from W3 and W4 [file 12966_2023_1551_MOESM6_ESM.docx]

**Additional file 6. Baseline characteristics of the study population from W3 and W4**

|  | | | |
| --- | --- | --- | --- |
|  | Overall (N = 855) | Male  (N = 332) | Female  (N = 523) |
| *Continuous variables: mean (SD)* |  |  |  |
| Sweet propensity^a^, score (range: 0 - 69.9) | 17.2 (10.5) | 18.0 (9.8) | 16.6 (10.8) |
| Fat propensity^a^, score (range: 0.9 - 64) | 25.4 (9.2) | 26.9 (9.5) | 24.5 (8.8) |
| Emotion-driven impulsiveness^ab^, score (range: 12 - 47) | 25.3 (7.3) | 23.9 (7.2) | 26.2 (7.2) |
| Psychosocial well-being^b^, score (range: 15 - 48) | 37.9 (5.6) | 38.8 (5.1) | 37.4 (5.8) |
| Age, years^a^ (range: 14.7 - 23.1) | 20.2 (0.9) | 20.2 (1.0) | 20.2 (0.8) |
| Media use, hours per week (range: 0 - 56; missing = 43) | 18.9 (11.7) | 23.4 (12.4) | 16.1 (10.2) |
| *Categorical variables: (%)* |  |  |  |
| Highest educational level of parents^c^ |  |  |  |
| high | 58.8 | 61.4 | 57.4 |
| low / medium | 40.4 | 38.0 | 41.9 |
| missing | 0.7 | 0.6 | 0.8 |
| Country |  |  |  |
| Central Europe | 39.2 | 42.5 | 37.1 |
| Northern Europe | 44.9 | 42.2 | 46.7 |
| Southern Europe | 15.9 | 15.4 | 16.3 |
| BMI^d^ |  |  |  |
| <25: thin/ normal weight | 77.7 | 76.2 | 78.6 |
| >=25: overweight / obesity | 22.3 | 23.8 | 21.4 |
| Physical activity  (sports club membership) |  |  |  |
| no | 65.4 | 67.5 | 64.1 |
| yes | 34.4 | 32.2 | 35.8 |
| missing | 0.2 | 0.3 | 0.2 |
| Sleep quality^d^ |  |  |  |
| 0 | 23.2 | 29.8 | 18.9 |
| 1 | 39.9 | 41.3 | 39.0 |
| 2-3 | 33.3 | 26.8 | 37.5 |
| missing | 3.6 | 2.1 | 4.6 |
| Baseline information derived from W3 (2013-2014) and W4 (2020-2021). Percentages may not add up to 100% due to rounding. ^a^ Information derived from W2 (2020-2021)  ^b^ Displayed as continuous variable but included as categorical variables in the main analysis ^c^ Based on International Standard Classification of Education Maximum (ISCED; maximum of both parents) ^d^ Displayed as categorical variables but included as continuous variables in the main analysis | | | |
