# Supplementary material for: The role of psychosocial well-being and emotion-driven impulsiveness in food choices of European adolescents
Source: Int J Behav Nutr Phys Act. 2024 Jan 2;21:1. doi: 10.1186/s12966-023-01551-w (PMC10759484; doi:10.1186/s12966-023-01551-w)
Supplement: Supplementary file 7 — Additional file 7. Estimated effects of psychosocial well-being and emotion-driven impulsiveness on average fat and sweet propensity; stratified by sex (at W3: Nmale: 970 and Nfemale: 1,095) [file 12966_2023_1551_MOESM7_ESM.docx]

**Additional file 13. Estimated effects of psychosocial well-being on average fat and sweet propensity using parametric regression standardisation (N = 2,065 at W3)**

|  |  | Outcome [MD (95%-CI)] | |
| --- | --- | --- | --- |
| Exposure | Category levels | Sweet propensity | Fat propensity |
| Psychosocial well-being | Ref. level: low |  |  |
|  | moderate | -0.22 (-1.25, 0.81) | -0.80 (-1.69, 0.08) |
|  | high | -1.52 (-2.64, -0.40) | -1.02 (-2.00, -0.03) |
| W2: Variables measured in 2009–2010; W3: Variables measured in 2013–2014 Ref. level: Reference level; MD: Mean Difference; 95% CI: 95% confidence interval  Exposure psychosocial well-being: adjusted for sweet or fat propensity score (depending on outcome), psychosocial well-being, age, highest educational level of parents, physical activity, sleep quality, and media use at W2; sex, country, and BMI at W3. Additional interaction terms similar to the ones in the causal mediation analysis were included. | | | |
