# Supplementary material for: The role of psychosocial well-being and emotion-driven impulsiveness in food choices of European adolescents
Source: Int J Behav Nutr Phys Act. 2024 Jan 2;21:1. doi: 10.1186/s12966-023-01551-w (PMC10759484; doi:10.1186/s12966-023-01551-w)
Supplement: Supplementary file 8 — Additional file 8. Estimated effects of psychosocial well-being and emotion-driven impulsiveness on average fat and sweet propensity; stratified by BMI (at W3: Nthin/normal weight: 1,530 and Noverweight/obesity: 535) [file 12966_2023_1551_MOESM8_ESM.docx]

**Additional file 14. Weights corresponding to each Super Learner algorithm obtained from the main analysis (Table 2) estimating the exposure or outcome mechanism.**

|  | Psychosocial well-being (ref. low category) | | | Emotion-driven impulsiveness  (ref. high category) | |
| --- | --- | --- | --- | --- | --- |
| Super Learner algorithm | Emotion-driven impulsiveness | Sweet propensity | Fat propensity | Sweet propensity | Fat propensity |
| To estimate exposure mechanism for moderate and high categories |  |  |  |  |  |
| Simple mean | 0 | 0 | 0 | 0 | 0 |
| Ranger^1^ | 0.17 | 0.24 | 0.22 | 0 | 0 |
| GLMNET^2^ | 0.84 | 0.76 | 0.79 | 0.67 | 0.76 |
| XGBOOST^3^ | 0 | 0 | 0 | 0.20 | 0.13 |
| SVM^4^ | 0 | 0 | 0 | 0.12 | 0.12 |
| To estimate outcome mechanism for moderate and high categories |  |  |  |  |  |
| Simple mean | 0.05 | 0 | 0.07 | 0 | 0.07 |
| GLM^5^ | 0.67 | 0.55 | 0.85 | 0.43 | 0.78 |
| Ranger^1^ | 0.03 | 0.20 | 0 | 0.22 | 0 |
| GLMNET^2^ | 0 | 0 | 0 | 0.19 | 0 |
| XGBOOST^3^ | 0.07 | 0 | 0.09 | 0 | 0.07 |
| SVM^4^ | 0.15 | 0.16 | 0 | 0.14 | 0.06 |
| earth^6^ | 0.04 | 0.09 | 0 | 0.01 | 0.03 |
| Due to the excessive computing time all machine learning algorithms were only used with default parameters  (see for reference: https://tlverse.org/sl3/reference/). ^1^ Random forests ^2^ Generalized linear models with elastic net regularization ^3^ Extreme gradient boosting ^4^ Support vector machines ^5^ Generalized linear models ^6^ Multivariate adaptive regression splines | | | | | |
