# Supplementary material for: The role of psychosocial well-being and emotion-driven impulsiveness in food choices of European adolescents
Source: Int J Behav Nutr Phys Act. 2024 Jan 2;21:1. doi: 10.1186/s12966-023-01551-w (PMC10759484; doi:10.1186/s12966-023-01551-w)
Supplement: Supplementary file 9 — Additional file 9. Estimated effects of psychosocial well-being and emotion-driven impulsiveness on fat and sweet propensity with variables measured at W3/W4 (N = 855 at W4, mean age = 20.2) [file 12966_2023_1551_MOESM9_ESM.docx]

**Additional file 15. Discussion on causal identification assumptions**

Regarding **conditional exchangeability**, we adjusted for a wide range of covariates based on an expert-constructed DAG. We, however, cannot fully rule out uncontrolled confounding due to other important factors such as personality type or food availability. Yet, we conducted a thorough assessment of potential covariates based on a framework for dietary behavior which was created by an interdisciplinary expert workgroup to summarize modifiability, relationship strength, and population-level effect of relevant determinants of dietary behavior^[[1]](#footnote-1)^. With respect to the **positivity** assumption, we identified potential positivity violations within the levels of psychosocial well-being, age, media use, sweet and fat propensity measured at W2. However, we did not detect major deviations from our main results. One major concern in social epidemiological research is the violation of the **consistency** assumption due to compound treatments such as in the case of income, education, or neighborhood characteristics^[[2]](#footnote-2)^. As we cannot rule out potential violations, we attempted to be as precise as possible when defining our hypothetical interventions; i.e. as described in the discussion section on actual interventions, a competence skill enhancement approach for psychosocial well-being^[[3]](#footnote-3)^ and a multidisciplinary behavioral intervention for emotion-driven impulsiveness^[[4]](#footnote-4)^.

1. Stok FM, Hoffmann S, Volkert D, Boeing H, Ensenauer R, Stelmach-Mardas M, et al. The DONE framework: Creation, evaluation, and updating of an interdisciplinary, dynamic framework 2.0 of determinants of nutrition and eating. PLOS ONE. 2017;12(2):e0171077. [↑](#footnote-ref-1)
2. Rehkopf DH, Glymour MM, Osypuk TL. The consistency assumption for causal inference in social epidemiology: When a rose is not a rose. Curr Epidemiol Rep. 2016;3(1):63-71. [↑](#footnote-ref-2)
3. Elfrink TR, Goldberg JM, Schreurs KM, Bohlmeijer ET, Clarke AM. Positive educative programme: A whole school approach to supporting children’s well-being and creating a positive school climate: a pilot study. Health Education. 2017;117:215-30. [↑](#footnote-ref-3)
4. Delgado-Rico E, Río-Valle JS, Albein-Urios N, Caracuel A, González-Jiménez E, Piqueras MJ, et al. Effects of a multicomponent behavioral intervention on impulsivity and cognitive deficits in adolescents with excess weight. Behavioural Pharmacology. 2012;23(5 and 6):609-15. [↑](#footnote-ref-4)
