# Supplementary material for: The role of psychosocial well-being and emotion-driven impulsiveness in food choices of European adolescents
Source: Int J Behav Nutr Phys Act. 2024 Jan 2;21:1. doi: 10.1186/s12966-023-01551-w (PMC10759484; doi:10.1186/s12966-023-01551-w)
Supplement: Supplementary file 11 — Additional file 11. Estimated effects of psychosocial well-being and emotion-driven impulsiveness on fat and sweet propensity with sociodemographic variables measured at W3 (N = 2,065 at W3) [file 12966_2023_1551_MOESM11_ESM.docx]

**Additional file 7. Estimated effects of psychosocial well-being and emotion-driven impulsiveness on average fat and sweet propensity; stratified by sex (at W3: N_male_: 970 and N_female_: 1,095)**

Stratified by sex, the strongest effect of high versus low psychosocial well-being on average sweet propensity was estimated for female participants [high: MD = -2.38, CI: -3.92 to -0.84]. Similarly, the strongest effects of low versus high levels of emotion-driven impulsiveness on average sweet [low: MD = -2.03, CI: -3.65 to -0.40] and fat [low: MD = -2.40, CI: -3.72 to -1.08] propensity occurred for female participants. When comparing high versus low psychosocial well-being on emotion-driven impulsiveness, the strongest effect was estimated again for female participants [high: MD = -5.16, CI: -6.23 to -4.09].

|  |  | Outcome [MD (95%-CI)] | | | | | |
| --- | --- | --- | --- | --- | --- | --- | --- |
| Exposure | Category levels | Emotion-driven impulsiveness | | Sweet propensity | | Fat propensity | |
| Psychosocial well-being | Ref. level: low | male | female | male | female | male | female |
|  | moderate | -2.43  (-3.74, -1.11) | -2.56  (-3.61, -1.51) | -0.57  (-2.27, 1.13) | -0.13  (-1.38, 1.64) | -1.28  (-2.99, 0.43) | -0.11  (-1.34, 1.12) |
|  | high | -4.76  (-6.05, -3.48) | -5.16  (-6.23, -4.09) | -0.35  (-2.11, 1.41) | -2.38  (-3.92, -0.84) | -1.27  (-2.93, 0.39) | -0.36  (-1.64, 0.91) |
| Emotion-driven impulsiveness | Ref. level: high |  |  |  |  |  |  |
|  | moderate | / | / | -1.22  (-2.95, 0.51) | -0.61  (-2.13, 0.91) | -0.76  (-0.86, 2.37) | -1.32  (-2.56, -0.08) |
|  | low | / | / | -1.69  (-3.40, 0.03) | -2.03  (-3.65, -0.40) | -1.08  (-2.70, 0.54) | -2.40  (-3.72, -1.08) |
| Variables measured in 2009–2010; W3: Variables measured in 2013–2014 Ref. level: Reference level; MD: Mean Difference; 95% CI: 95% confidence interval  Exposure psychosocial well-being: adjusted for sweet or fat propensity (depending on outcome), psychosocial well-being, age, highest educational level of parents, physical activity, sleep quality, and media use at W2; country and BMI at W3 Exposure emotion-driven impulsiveness: adjusted for sweet or fat propensity (depending on outcome), psychosocial well-being, age, highest educational level of parents, physical activity, sleep quality, and media use at W2; psychosocial well-being, country, and BMI at W3 | | | | | | | |
