# Supplementary material for: The role of psychosocial well-being and emotion-driven impulsiveness in food choices of European adolescents
Source: Int J Behav Nutr Phys Act. 2024 Jan 2;21:1. doi: 10.1186/s12966-023-01551-w (PMC10759484; doi:10.1186/s12966-023-01551-w)
Supplement: Supplementary file 12 — Additional file 12. Estimated effects of psychosocial well-being and emotion-driven impulsiveness on average fat and sweet propensity in subgroup without positivity violations (N = 1,954 at W3) [file 12966_2023_1551_MOESM12_ESM.docx]

**Additional file 8. Estimated effects of psychosocial well-being and emotion-driven impulsiveness on average fat and sweet propensity; stratified by BMI (at W3: N_thin/normal weight_: 1,530 and N_overweight/obesity_: 535)**

When stratified by BMI, the strongest effects of high versus low psychosocial well-being on average sweet and fat propensity occurred in adolescents with overweight or obesity. Similarly, the strongest effects of low versus high levels of emotion-driven impulsiveness on average sweet [low: MD = -3.22, CI: -5.55 to -0.89] and fat [low: MD = -2.53, CI: -4.69 to -0.37] propensity occurred in adolescents with overweight and obesity. When comparing different levels of psychosocial well-being on emotion-driven impulsiveness, the strongest effects occurred in adolescents with overweight or obesity for moderate psychosocial well-being [moderate: MD = -3.18, CI: -4.76 to -1.61] and in thin or normal weight adolescents for high psychosocial well-being [high: MD = -5.14, CI: -6.06 to -4.22].

|  |  | Outcome [MD (95%-CI)] | | | | | |
| --- | --- | --- | --- | --- | --- | --- | --- |
| Exposure | Category levels | Emotion-driven impulsiveness | | Sweet propensity | | Fat propensity | |
| Psychosocial well-being | Ref. level: low | thin/normal weight | overweight/obesity | thin/normal weight | overweight/obesity | thin/normal weight | overweight/obesity |
|  | moderate | -2.42  (-3.36, -1.47) | -3.18  (-4.76, -1.61) | 0.19  (-1.16, 1.53) | -1.42  (-3.49, 0.65) | -0.25  (-1.43, 0.94) | -0.73  (-2.70, 1.24) |
|  | high | -5.14  (-6.06, -4.22) | -4.81  (-6.59, -3.02) | -1.01  (-2.40, 0.37) | -2.06  (-4.27, 0.16) | -0.47  (-1.66, 0.73) | -0.25  (-2.41, 1.90) |
| Emotion-driven impulsiveness | Ref. level: high |  |  |  |  |  |  |
|  | moderate | / | / | -1.01  (-2.38, 0.35) | -1.46  (-3.57, 0.66) | -0.28  (-1.42, 0.85) | -1.10  (-3.06, 0.87) |
|  | low | / | / | -1.90  (-3.26, -0.54) | -3.22  (-5.55, -0.89) | -1.65  (-2.81, -0.49) | -2.53  (-4.69, -0.37) |
| W2: Variables measured in 2009–2010; W3: Variables measured in 2013–2014 Ref. level: Reference level; MD: Mean Difference; 95% CI: 95% confidence interval  Exposure psychosocial well-being: adjusted for sweet or fat propensity (depending on outcome), psychosocial well-being, age, highest educational level of parents, physical activity, sleep quality, and media use at W2; sex and country at W3 Exposure emotion-driven impulsiveness: adjusted for sweet or fat propensity (depending on outcome), psychosocial well-being, age, highest educational level of parents, physical activity, sleep quality, and media use from W2; psychosocial well-being, sex, and country at W3 | | | | | | | |
