# Supplementary material for: The role of psychosocial well-being and emotion-driven impulsiveness in food choices of European adolescents
Source: Int J Behav Nutr Phys Act. 2024 Jan 2;21:1. doi: 10.1186/s12966-023-01551-w (PMC10759484; doi:10.1186/s12966-023-01551-w)
Supplement: Supplementary file 13 — Additional file 13. Estimated effects of psychosocial well-being on average fat and sweet propensity using parametric regression standardisation (N = 2,065 at W3) [file 12966_2023_1551_MOESM13_ESM.docx]

**Additional file 9. Estimated effects of psychosocial well-being and emotion-driven impulsiveness on fat and sweet propensity with variables measured at W3/W4 (N = 855 at W4, mean age = 20.2)**

|  |  | Outcome [MD (95%-CI)] | | | |
| --- | --- | --- | --- | --- | --- |
| Exposure | Category levels | Emotion-driven impulsiveness (W4) | Sweet propensity (W4) | Fat propensity (W4) | |
| Psychosocial well-being (W3) | Ref. level: low |  |  |  | |
|  | moderate | -2.43 (-3.69, -1.16) | -0.21 (-2.06, 1.64) | 0.22 (-1.39, 1.84) |  |
|  | high | -4.66 (-5.94, -3.38) | -1.44 (-3.30, 0.42) | 0.27 (-1.39, 1.92) | |
| Emotion-driven impulsiveness (W4) | Ref. level: high |  |  |  | |
|  | moderate | / | -1.82 (-3.54, -0.09) | 0.31 (-1.09, 1.71) | |
|  | low | / | -3.13 (-5.04, -1.22) | 0.42 (-1.97, 1.14) | |
| W2: Variables measured in 2009–2010; W3: Variables measured in 2013–2014;  W4: Variables measured in 2021–2022 Ref. level: Reference level; MD: Mean Difference; 95% CI: 95% confidence interval  Exposure psychosocial well-being: adjusted for sweet or fat propensity score (depending on outcome), psychosocial well-being, age, highest educational level of parents, physical activity, sleep quality, and media use at W2; sex, country, and BMI at W3  Exposure emotion-driven impulsiveness: adjusted for emotion-driven impulsiveness, psychosocial well-being, age, sex, highest educational level of parents, physical activity, sleep quality, and media use at W2; psychosocial well-being, sex, country, and BMI at W3 | | | | | |
