# Supplementary material for: The role of psychosocial well-being and emotion-driven impulsiveness in food choices of European adolescents
Source: Int J Behav Nutr Phys Act. 2024 Jan 2;21:1. doi: 10.1186/s12966-023-01551-w (PMC10759484; doi:10.1186/s12966-023-01551-w)
Supplement: Supplementary file 14 — Additional file 14. Weights corresponding to each Super Learner algorithm obtained from the main analysis (Table 2) estimating the exposure or outcome mechanism [file 12966_2023_1551_MOESM14_ESM.docx]

**Additional file 10. Estimated effects of psychosocial well-being and emotion-driven impulsiveness on fat and sweet propensity with health-related variables measured at W3 (N = 2,065 at W3)**

|  |  | Outcome [MD (95%-CI)] | | |
| --- | --- | --- | --- | --- |
| Exposure | Category levels | Emotion-driven impulsiveness | Sweet propensity | Fat propensity |
| Psychosocial well-being | Ref. level: low |  |  |  |
|  | moderate | -1.99 (-2.83, -1.14) | 0.30 (-0.91, 1.51) | -0.36 (-1.37, 0.64) |
|  | high | -4.18 (-5.08, -3.29) | -0.35 (-1.64, 0.95) | -0.41 (-1.52, 0.70) |
| Emotion-driven impulsiveness | Ref. level: high |  |  |  |
|  | moderate | / | -0.74 (-1.88, 0.40) | -0.16 (-1.15, 0.83) |
|  | low | / | -1.68 (-2.87, -0.48) | -1.52 (-2.57, -0.46) |
| W2: Variables measured in 2009–2010; W3: Variables measured in 2013–2014 Ref. level: Reference level; MD: Mean Difference; 95% CI: 95% confidence interval  Exposure psychosocial well-being: adjusted for sweet or fat propensity score (depending on outcome), psychosocial well-being, age, and highest educational level of parents at W2; physical activity, sleep quality, media use, sex, country, and BMI at W3  Exposure emotion-driven impulsiveness: adjusted for sweet or fat propensity score (depending on outcome), psychosocial well-being, age, highest educational level of parents, country, physical activity, sleep quality, and media use at W2; psychosocial well-being, physical activity, sleep quality, media use, sex, country, and BMI at W3 | | | | |
