# Supplementary material for: The role of psychosocial well-being and emotion-driven impulsiveness in food choices of European adolescents
Source: Int J Behav Nutr Phys Act. 2024 Jan 2;21:1. doi: 10.1186/s12966-023-01551-w (PMC10759484; doi:10.1186/s12966-023-01551-w)
Supplement: Supplementary file 15 — Additional file 15. Discussion on causal identification assumptions [file 12966_2023_1551_MOESM15_ESM.docx]

**Additional file 11. Estimated effects of psychosocial well-being and emotion-driven impulsiveness on fat and sweet propensity with sociodemographic variables measured at W3 (N = 2,065 at W3)**

|  |  | Outcome [MD (95%-CI)] | | |
| --- | --- | --- | --- | --- |
| Exposure | Category levels (ref. low) | Emotion-driven impulsiveness | Sweet propensity | Fat propensity |
| Psychosocial well-being | Ref. level: low |  |  |  |
|  | moderate | -2.49 (-3.31, -1.67) | -0.11 (-1.24, 1.03) | -0.60 (-1.59, 0.40) |
|  | high | -4.88 (-5.71, -4.04) | -1.29 (-2.47, -0.10) | -0.82 (-1.83, 0.19) |
| Emotion-driven impulsiveness | Ref. level: high |  |  |  |
|  | moderate | / | -0.92 (-2.06, 0.24) | -0.26 (-1.28, -0.75) |
|  | low | / | -1.97 (-3.15, -0.79) | -1.85 (-2.90, -0.80) |
| W2: Variables measured in 2009–2010; W3: Variables measured in 2013–2014 Ref. level: Reference level; MD: Mean Difference; 95% CI: 95% confidence interval  Exposure psychosocial well-being: adjusted for sweet or fat propensity score (depending on outcome), psychosocial well-being, physical activity, sleep quality, and media use at W2; age, sex, highest educational level of parents, country, and BMI at W3  Exposure emotion-driven impulsiveness: adjusted for sweet or fat propensity score (depending on outcome), psychosocial well-being, age, highest educational level of parents, country, physical activity, sleep quality, and media use at W2; psychosocial well-being, age, sex, highest educational level of parents, country, and BMI at W3 | | | | |
