# Supplementary material for: The role of psychosocial well-being and emotion-driven impulsiveness in food choices of European adolescents
Source: Int J Behav Nutr Phys Act. 2024 Jan 2;21:1. doi: 10.1186/s12966-023-01551-w (PMC10759484; doi:10.1186/s12966-023-01551-w)
Supplement: Supplementary file 16 — Supplementary Material 16 [file 12966_2023_1551_MOESM16_ESM.docx]

**Additional file 12. Estimated effects of psychosocial well-being and emotion-driven impulsiveness on average fat and sweet propensity in subgroup without positivity violations (N = 1,954 at W3)**

|  |  | Outcome [MD (95%-CI)] | | |
| --- | --- | --- | --- | --- |
| Exposure | Category levels | Emotion-driven impulsiveness | Sweet propensity | Fat propensity |
| Psychosocial well-being | Ref. level: low |  |  |  |
|  | moderate | -2.36 (-3.19, -1.54) | -0.17 (-1.36, 1.01) | -0.34 (-1.37, 0.69) |
|  | high | -4.82 (-5.64, -4.00) | -1.13 (-2.35, 0.10) | -0.46 (-1.49, 0.57) |
| Emotion-driven impulsiveness | Ref. level: high |  |  |  |
|  | moderate | / | -1.00 (-2.19, 0.20) | -0.63 (-1.65, 0.40) |
|  | low | / | -2.09 (-3.33, -0.84) | -1.87 (-2.94, -0.79) |
| W2: Variables measured in 2009–2010; W3: Variables measured in 2013–2014 Ref. level: Reference level; MD: Mean Difference; 95% CI: 95% confidence interval  Exposure psychosocial well-being: adjusted for sweet or fat propensity score (depending on outcome), psychosocial well-being, age, highest educational level of parents, physical activity, sleep quality, and media use at W2; sex, country, and BMI at W3  Exposure emotion-driven impulsiveness: adjusted for sweet or fat propensity score (depending on outcome), psychosocial well-being, age, highest educational level of parents, physical activity, sleep quality, and media use at W2; psychosocial well-being, sex, country, and BMI at W3 | | | | |
